# Anthrax toxin component, Protective Antigen, protects insects from bacterial infections

**DOI:** 10.1371/journal.ppat.1008836

**Published:** 2020-08-31

**Authors:** Saleem Alameh, Gloria Bartolo, Summer O’Brien, Elizabeth A. Henderson, Leandra O. Gonzalez, Stella Hartmann, Christopher P. Klimko, Jennifer L. Shoe, Christopher K. Cote, Laurence K. Grill, Anastasia Levitin, Mikhail Martchenko Shilman

**Affiliations:** 1 Henry E. Riggs School of Applied Life Sciences, Keck Graduate Institute, Claremont, California, United States of America; 2 Bacteriology Division, US Army Medical Research Institute of Infectious Diseases, Fort Detrick, Frederick, Maryland, United States of America; Pennsylvania State University, UNITED STATES

## Abstract

Anthrax is a major zoonotic disease of wildlife, and in places like West Africa, it can be caused by *Bacillus anthracis* in arid nonsylvatic savannahs, and by *B*. *cereus* biovar *anthracis* (Bcbva) in sylvatic rainforests. Bcbva-caused anthrax has been implicated in as much as 38% of mortality in rainforest ecosystems, where insects can enhance the transmission of anthrax-causing bacteria. While anthrax is well-characterized in mammals, its transmission by insects points to an unidentified anthrax-resistance mechanism in its vectors. In mammals, a secreted anthrax toxin component, 83 kDa Protective Antigen (PA_83_), binds to cell-surface receptors and is cleaved by furin into an evolutionary-conserved PA_20_ and a pore-forming PA_63_ subunits. We show that PA_20_ increases the resistance of *Drosophila* flies and *Culex* mosquitoes to bacterial challenges, without directly affecting the bacterial growth. We further show that the PA_83_ loop known to be cleaved by furin to release PA_20_ from PA_63_ is, in part, responsible for the PA_20_-mediated protection. We found that PA_20_ binds directly to the Toll activating peptidoglycan-recognition protein-SA (PGRP-SA) and that the Toll/NF-κB pathway is necessary for the PA_20_-mediated protection of infected flies. This effect of PA_20_ on innate immunity may also exist in mammals: we show that PA_20_ binds to human PGRP-SA ortholog. Moreover, the constitutive activity of Imd/NF-κB pathway in MAPKK Dsor1 mutant flies is sufficient to confer the protection from bacterial infections in a manner that is independent of PA_20_ treatment. Lastly, *Clostridium septicum* alpha toxin protects flies from anthrax-causing bacteria, showing that other pathogens may help insects resist anthrax. The mechanism of anthrax resistance in insects has direct implications on insect-mediated anthrax transmission for wildlife management, and with potential for applications, such as reducing the sensitivity of pollinating insects to bacterial pathogens.

## Author summary

Gram-positive *Bacillus anthracis* and *B*. *cereus* biovar *anthracis* cause anthrax in mammals by secreting anthrax toxin. Recent studies report the devastating ecological effects anthrax has on the wildlife in areas like tropical rainforests, where in the past 26 years, anthrax had been implicated in 38% of wildlife mortality, and where insects can act as the vectors for anthrax transmission. The anthrax-resistance mechanism in its insect vectors has not been identified. We discovered that one of anthrax toxin components reduces the sensitivity of insects to anthrax-causing bacilli and other bacteria, and that insect immunity Toll/NF-κB and Imd/NF-κB pathways play a role in this toxin-mediated protection. The toxin-mediated protection of insects would benefit the anthrax-causing bacteria by extending the lifespan of vectors and enhancing the pathogen transmission. Anthrax toxin component activation of NF-κB has also been found to exists in humans. Moreover, the activation of the innate NF-κB-dependent immunity by this anthrax toxin component has potential implications in protecting pollinating insects from bacterial pathogens.

## Introduction

Anthrax is a major zoonotic disease of wildlife caused by the Gram-positive bacterium *Bacillus anthracis* or by the closely related *B*. *cereus* biovar *anthracis* (Bcbva), which combines the chromosomal background of *B*. *cereus* with the toxin-encoding pXO1 and the capsule-encoding pXO2 plasmids of *B*. *anthracis* [1]. Bcbva causes “sylvatic anthrax”, a prevalent and persistent cause of death in a broad range of mammalian hosts in the rainforest ecosystem of Taï National Park (TNP), Côte d'Ivoire. Sylvatic anthrax was responsible for as much as 38% of wildlife mortality observed over 26 years in TNP and is predicted to accelerate the decline and possibly result in the local extinction of chimpanzee populations [2, 3]. Moreover, viable spores of Bcbva were detected in blow flies sampled inside and outside the chimpanzee habitats in TNP [2]. In addition to TNP, Bcbva has been isolated from dead or sick mammals in other West and Central Africa regions, such as Cameroon, Central African Republic, and Democratic Republic of Congo [4], as well as in regions outside of Africa, such as Texas and Louisiana, USA [5].

In contrast, *B*. *anthracis*, which is distributed globally [6], causes “classical anthrax” that is most commonly observed in arid nonsylvatic ecosystems, such as African savannahs in Krüger National Park [7], South Africa and Etosha National Park, Namibia [8, 9]. In these ecosystems, major anthrax outbreaks display strong seasonal variation, where the mortality coincides with rainfall cycles and the wet season [7–9]. In these nonsylvatic ecosystems, it has also been suggested that flies spread *B*. *anthracis* spores from carcasses through the environment, potentially contributing to anthrax transmission [10, 11].

In nature, insects can serve as the vectors for mechanical anthrax transmission to humans, cattle, and the mammalian wildlife [2, 6, 10, 12–14]. Biting flies have been shown to acquire anthrax-causing bacilli from infected animals and directly transmit them to other mammals [14]. Similarly, non-biting flies feed on the bodily fluids of infected carcasses and deposit contaminated feces or vomit on nearby vegetation, creating a reservoir for herbivores to potentially contract anthrax while grazing [7]. Other insects, such as mosquitoes and ticks, may also contribute to anthrax transmission [15–17], raising the interesting question as to how insect vectors tolerate anthrax-mediated toxicity.

Insects depend on their innate immunity for protection against pathogens. During an infection, insects produce antimicrobial peptides (AMP), while circulating hemocytes phagocytose invading microbes [18]. Two classical signaling pathways, the Toll and the immune deficiency (Imd) pathways, mediate broad-spectrum AMP responses. The Toll pathway is primarily activated by lysine (lys)-type peptidoglycan found in most Gram-positive bacterial cell walls, which interacts with insect PGRP-SA [19]. In contrast, the Imd pathway is mostly activated in response to diaminopimelate (dap)-type peptidoglycans found in all Gram-negative bacteria and Gram-positive *Bacillus* species, which interact with insect PGRP-LC and PGRP-LE [20]. Interestingly, it was recently shown that the sensing of the bacterial cell wall type is not as pathway-specific as previously thought: as long as the bacterial wall is accessible, both Toll and Imd pathways can detect lys- and dap-peptidoglycan, since dap is a derivative of lys [21]. Activation of these pathways leads to the induction of nuclear factor-κB (NF-κB) transcription factors, which promote the transcription of AMPs, whose expression levels could be regulated by both pathways, and with many of them showing broad-spectrum antimicrobial activity [22–25].

Both Bcbva and *B*. *anthracis* secrete the toxin encoded by plasmid pXO1, which can ultimately kill mammals [2]. The pXO1 plasmid encodes three toxin components that injure mammalian cells: protective antigen (PA), lethal factor (LF), and edema factor (EF) [26]. During anthrax infection, 83 kDa PA (PA_83_) binds to mammalian cell-surface receptors and is cleaved by host furin proteases into 20 kDa (PA_20_) and 63 kDa (PA_63_) subunits [27]. Following the cleavage, PA_20_ dissociates from PA_63_, and receptor-bound PA_63_ multimerize, forming a pre-pore, which binds EF and LF before endocytosis [28, 29]. In the acidified endosome, the PA_63_ pore allows EF and LF to escape into the cytosol to exert their deleterious effects [30]. Although PA_20_ is evolutionary-conserved [31], its function is poorly understood: in addition to blocking the multimerization of PA_83_, it may play a role in activating adaptive and innate immunity responses. During anthrax, the major adaptive immune response is mounted against PA_83_, and several epitopes within PA_20_ have been identified in PA_83_-immunized mammalian hosts [32–35]. In addition to inducing adaptive immunity, PA_20_ may also activate innate immunity: the host cell exposure to PA_83_ or PA_20_ was shown to activate NF-κB and pro-inflammatory cytokines, such as Interleukins [36–38].

It was previously observed that several insect species, such as blow flies, horn flies, house flies, and mosquitoes are insensitive to *B*. *anthracis* oral exposure [11, 15, 17, 39]. In contrast, insects are sensitive to non-*anthracis* strains of closely related *B*. *cereus* [40–42]. Therefore, we hypothesize that anthrax toxin components may contribute to insects’ resistance to bacilli, as insects feed on anthrax-infected mammals or their carcasses. Previous studies have established *Drosophila melanogaster* as a fly model to study the effects of genetically expressed LF and EF [43]. Here, we discovered that when administered orally, PA_20_ reduces the sensitivity of *Drosophila* flies and *Culex* mosquitoes to insecticidal bacteria, and further study the mechanism by which insects resist anthrax. We propose that this toxin-dependent phenomenon may provide insects with a counterbalance to the adverse effects of insecticidal bacteria, thereby facilitating the spread of anthrax.

## Results

### PA_83_ protects infected flies from *B. cereus* infection

*B*. *anthracis* and Bcbva belong to a *sensu lato* group of bacteria, called *B*. *cereus*, which comprises seven closely related species that includes *B*. *cereus sensu stricto* [44] (referred to herein as *B*. *cereus*). The phylogenetic cluster that includes *B*. *anthracis* and Bcbva also includes *B*. *cereus* strain ATCC 10987 [44, 45]. While the chromosome of *B*. *cereus* strain ATCC 10987 shares close homology to the *B*. *anthracis* genome (complete nucleotide sequence identity of 94%) and the Bcbva genome, this strain lacks anthrax toxin orthologues on its pXO1-like plasmid pBc10987 [44–46]. This, together with the fact that specific isolates of *B*. *cereus* cause sylvatic anthrax in tropical rainforests [1–4, 46], presented an opportunity to test the effects of anthrax toxin components on the sensitivity of *Drosophila* to this Bcbva-related bacterium.

**Fig 1:**
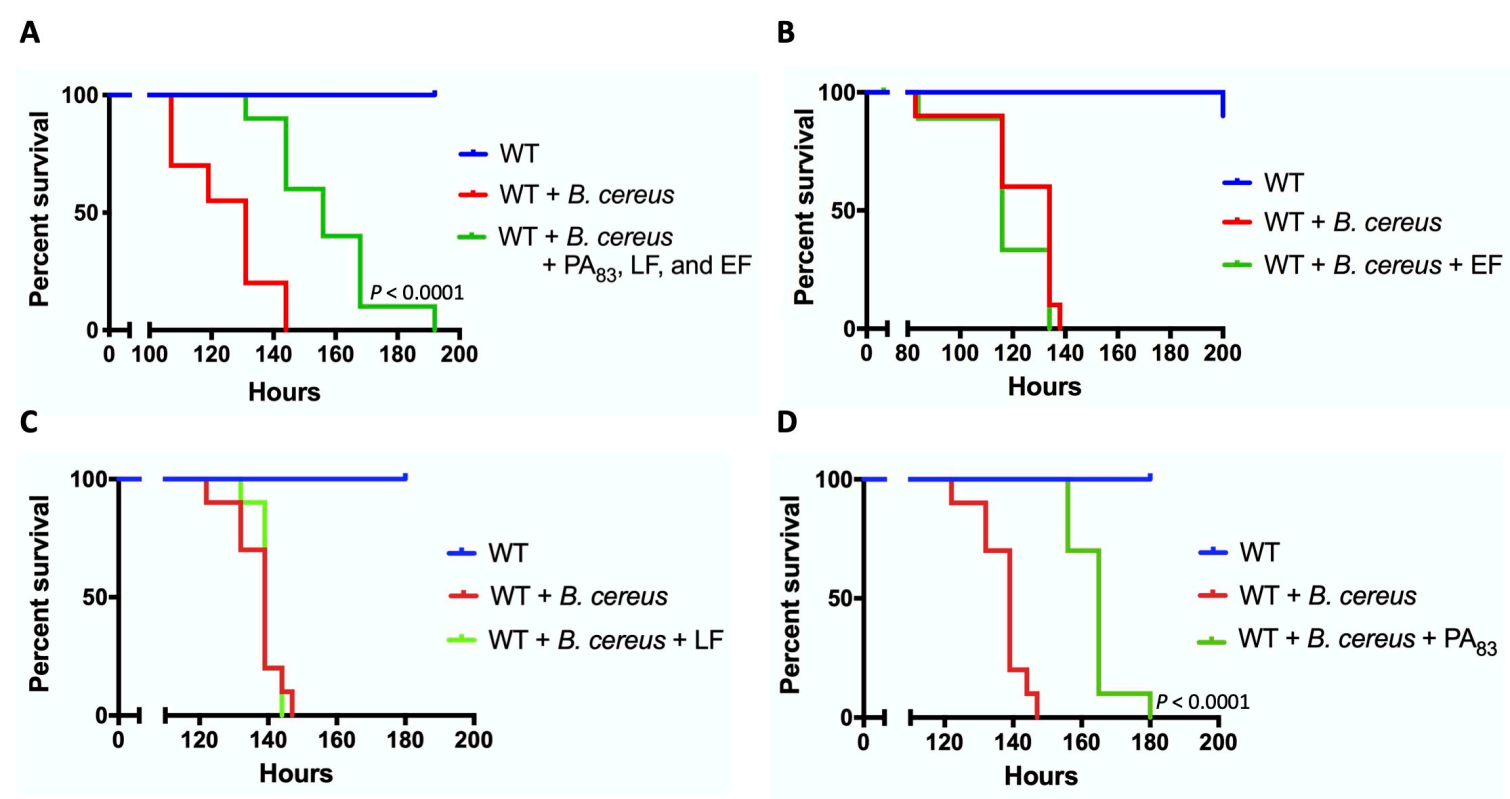
Anthrax toxin component PA_83_ protects *Drosophila* from *Bacillus cereus*. The discovery of PA-mediated reduction in sensitivity of flies to *B*. *cereus* infection. Male Oregon-R wild type (WT) flies were challenged with *B*. *cereus* in the absence or presence of one or more anthrax toxin components, including a combination of all toxin components (A), EF (B), LF (C), and PA_83_ (D). *P* values indicate statistical significance compared to the bacteria-only condition.

When feeding on anthrax-infected mammals, insects can be exposed to anthrax toxin components. We observed that the oral exposure of flies to a mix of purified components PA_83_, LF, and EF in the absence of the bacterial challenge did not alter their survival (S1 Fig). In the presence of the anthrax toxin components, flies showed increased survival time (quantified as the increased median survival time) to *B*. *cereus* infection by 25 hours, *P* < 0.0001 (Fig 1A). While neither EF nor LF affected the sensitivity of flies to *B*. *cereus* (Fig 1B and 1C), PA_83_ alone delayed the death of infected flies (delay of the median survival time by 26 hours, *P* < 0.0001) (Fig 1D). In addition to flies aged 4–5 days (Fig 1), the protective effect of PA_83_ is also observed in flies of various ages (delay of the median survival time by 34 hours, *P* < 0.0001) (S2 Fig). These results show that protein regions located within PA_83_ cause the delay in bacilli-induced death in *Drosophila*.

### The effect of PA 1β_13_−1α_1_ loop and PA_20_ on insects’ sensitivity to bacterial infections

**Fig 2:**
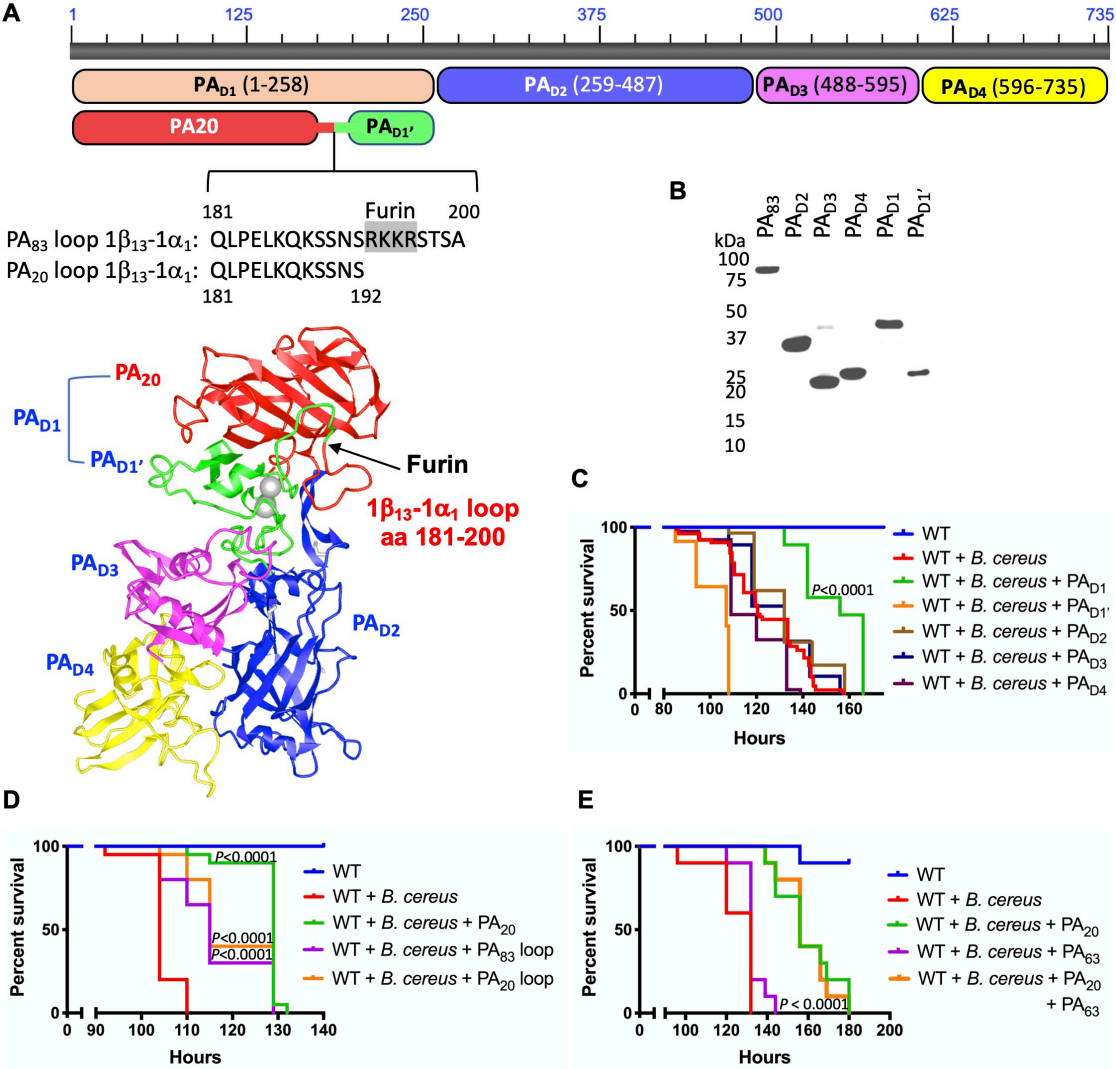
Structure-function analysis of PA_83_. (A) Domain organization in the linear and tertiary structure or PA_83_. The domains and the corresponding amino acids are indicated in the linear structure of PA_83_. The full tertiary structure of monomeric PA_83_ is shown using iCn3D (Protein Data Bank ID: 4H2A). The regions in blue, purple, and yellow represent the domains 2, 3, and 4 portions of PA_63_, while the green region represents the portion of domain 1 (aka domain 1’) that remains part of PA_63_ after furin cleavage, and the red region represents PA_20_. Furin cleavage occurs in the 1β_13_−1α_1_ loop composed of amino acids 181–200. The amino acid sequence of the PA_83_ loop 1β_13_−1α_1_ connecting PA_20_ to domain 1’ is also shown. After furin cleavage (the furin cleavage site is shaded gray), amino acids 181–192 are retained by PA_20_ (shown in the second line labeled PA_20_ 1β_13_−1α_1_ loop). (B) Western blot showing the ability of the polyclonal anti-PA_83_ antibody to recognize recombinantly expressed and purified PA domains 1, 1’, 2, 3, and 4. (C) The effect of the PA domains 1, 1’, 2, 3, and 4 (PA_D1_, PA_D1’_, PA_D2_, PA_D3_, and PA_D4_) on the sensitivity of *Drosophila* to *B*. *cereus*. WT flies of both genders were pooled and challenged with *B*. *cereus* with or without individual PA domains. (D) The effect of the 1β_13_−1α_1_ loop on the sensitivity of *Drosophila* to *B*. *cereus*. WT flies were challenged with *B*. *cereus* with or without PA_20_, PA_83_ loop 181–200, or PA_20_ loop 181–192. (E) WT flies were challenged with *B*. *cereus* in the absence or presence of furin-processed PA_20_, PA_63_, or their combination. *P* as in Fig 1.

PA_83_ contains four functional domains (Fig 2A): the host receptor-binding domains 2 and 4 (PA_D2_ and PA_D4_), the multimerization domain 3 (PA_D3_), and the PA_20_-containing domain 1 (PA_D1_) [29]. After furin cleavage, the portion of PA_D1_ that remains on PA_63_ is called domain 1’ (PA_D1’_). To investigate the region within PA_83_ responsible for the protection of infected flies, we recombinantly expressed all PA_83_ domains (Fig 2B). We observed that PA_20_-containing PA_D1,_ but not PA_D1’_, PA_D2_, PA_D3_, nor PA_D4_, protected flies from *B*. *cereus* infection (delay of the median survival time by 37 hours, *P* < 0.0001) (Fig 2C).

Like insects, plants rely only on innate immunity to protect them from pathogens. In plants, programmed cell death, or necrotic lesions, often occur at the site of infection to prevent the systemic spread of invading microorganisms [47]. Previously we observed that amino acids 181–200 of PA_83_ caused necrotic lesions in *Nicotiana benthamiana* [48], suggesting that they trigger an innate immune response in plants. This peptide forms an unstructured loop (between β-sheet 13 and α-helix 1, referred to as the “1β_13_−1α_1_ loop”) within domain 1 of PA_83_ [29], which contains the furin-cleavage site [49] (Fig 2A). The loop residues 181–192 remain on PA_20_ after furin processing. We assessed the ability of the entire loop (residues 181–200) and the furin-cleaved portion (residues 181–192) of the 1β_13_−1α_1_ loop to reduce the sensitivity of flies to bacterial infections. To some degree, both peptides delayed the death of *B*. *cereus*-infected flies (delay of the median survival time 11 hours, where some flies survive for 29 hours longer, *P* < 0.0001) (Fig 2D) while also not directly altering bacterial growth (S3 Fig). Therefore, the residues within the PA_20_ 1β_13_−1α_1_ loop are, in part, responsible for the PA_83_-mediated reduction in the sensitivity of flies to bacterial infections.

When feeding on anthrax-infected mammals, insects can be exposed to PA in its native PA_83_ state, as well as in the furin-cleaved state, PA_63_ and PA_20_. Further testing revealed that within PA_83_, it was PA_20_ and not PA_63_ responsible for the reduction in fly sensitivity to *B*. *cereus*: PA_20_ protected infected flies without and with PA_63_ (delay of the median survival time by 24 hours, *P* < 0.0001) (Fig 2E).

### PA_20_ protection is broad-spectrum and insect-directed

**Fig 3:**
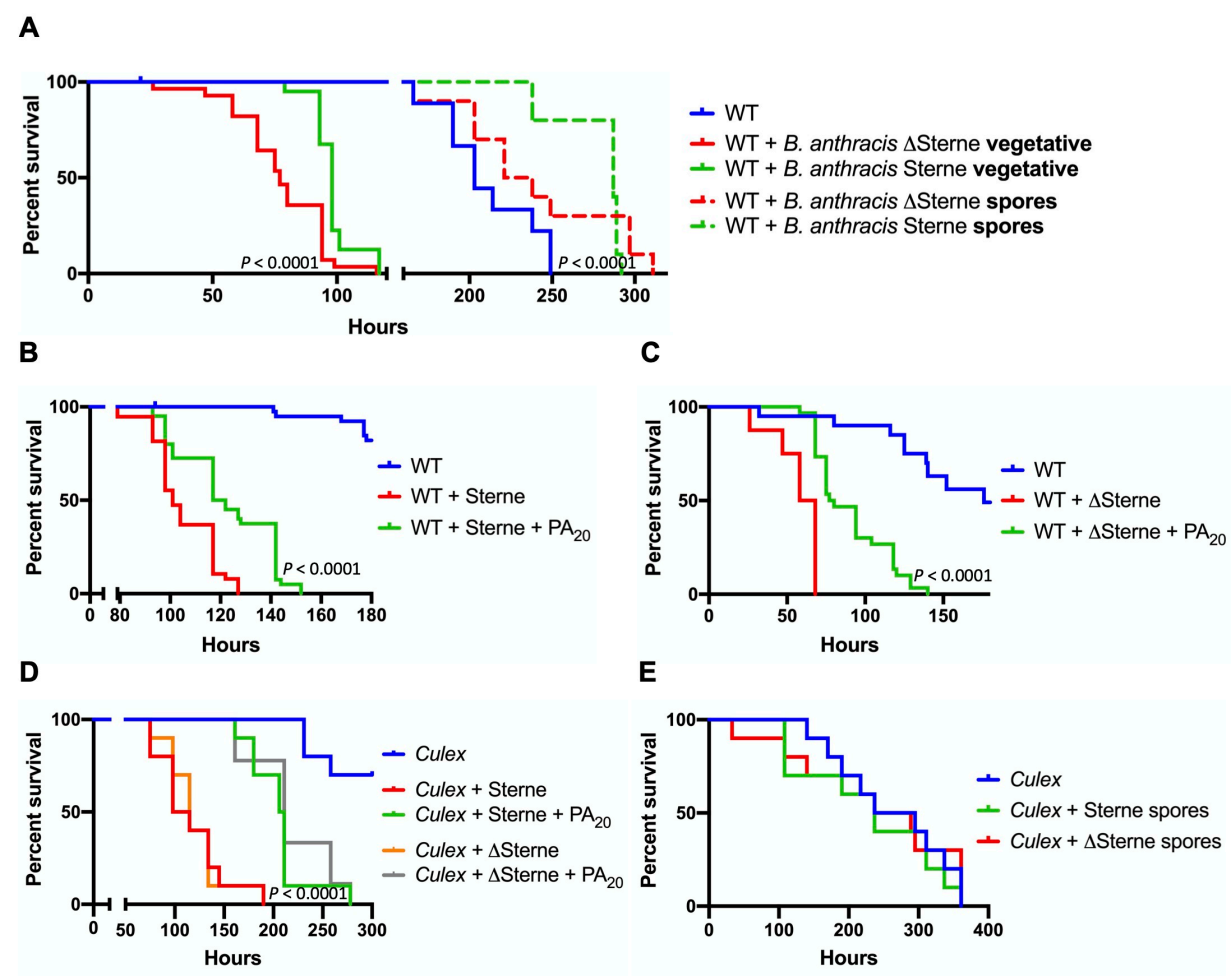
PA_20_ protects *Drosophila* from *Bacillus anthracis*. (A) WT flies were orally challenged with *B*. *anthracis* Sterne and plasmid-cured ΔSterne strains in vegetative and spore states. *P* values in (A) indicate the statistical significance of Sterne vs. ΔSterne for vegetative and spore states. (B-C) WT flies were orally challenged with vegetative cells of the *B*. *anthracis* Sterne (B) and ΔSterne (C) strains with or without PA_20_. *P* as in Fig 1. (D) Male *Culex quinquefasciatus* (*Culex*) were orally challenged with vegetative *B*. *anthracis* Sterne or ΔSterne strains with or without PA_20_. *P* as in Fig 1. (E) *C*. *quinquefasciatus* (*Culex*) mosquitoes were orally challenged with spores of *B*. *anthracis* Sterne or ΔSterne strains.

We tested the sensitivity of *D*. *melanogaster* to oral administration of the toxigenic Sterne strain of *B*. *anthracis* harboring the pXO1 plasmid, and the ΔSterne strain, a pXO1-cured derivative of the Sterne strain. While flies were sensitive to both *B*. *anthracis* strains in the vegetative state, surprisingly, the Sterne strain was less pathogenic than the toxin-negative ΔSterne strain (delay of the median survival time by 21 hours, *P* < 0.0001) (Fig 3A). Moreover, we investigated the sensitivity of flies to spores of the same *B*. *anthracis* strains. Interestingly, while uninfected flies feeding solely on sucrose can survive for 10–14 days, the oral exposure to *B*. *anthracis* spores extended the longevity of flies, with the Sterne strain extending the life significantly longer than the ΔSterne strain (delay of the median survival time by 57 hours, *P* < 0.0001) (Fig 3A). These data show that the toxin-containing strain of *B*. *anthracis* is less pathogenic to flies and extends the survival of flies, compared to the toxin-negative strain of bacteria.

When feeding on anthrax-infected mammals, insects can be exposed to pXO1-containing bacteria, such as Bcbva and *B*. *anthracis* cells, as well as secreted toxins components, including PA_20_. We show that exogenously added PA_20_ protects flies from the Sterne strain (Fig 3B) and ΔSterne strain (Fig 3C). PA_20_ delayed the median survival time of Sterne strain infected flies by 19 hours (*P* < 0.0001) and of ΔSterne strain infected flies by 16 hours (*P* < 0.0001), although some flies were protected by as long as 25–70 hours.

Mosquitoes may also contribute to anthrax transmission [15, 17] and were previously found to be insensitive to anthrax toxin [50]. We tested whether PA_20_-mediated protection occurs in *B*. *anthracis*-infected mosquitoes, *Culex quinquefasciatus*. Interestingly while mosquitoes were sensitive to the vegetative Sterne and ΔSterne strains of *B*. *anthracis*, in contrast to *Drosophila*, their sensitivity was similar to both strains (Fig 3D). Moreover, in contrast to our fruit fly experiments, oral exposure to *B*. *anthracis* spores did not affect the longevity of mosquitoes (Fig 3E). Nevertheless, PA_20_ significantly protected *Culex* from both strains of bacteria and delayed the median survival time of Sterne strain infected mosquitoes by 102 hours (*P* < 0.0001) (Fig 3D) and of ΔSterne strain infected mosquitoes by 96 hours (*P* < 0.0001) (Fig 3D). Collectively, these results show that PA_20_ causes the delay of *B*. *anthracis*-induced death in *Drosophila* and *Culex*.

**Fig 4:**
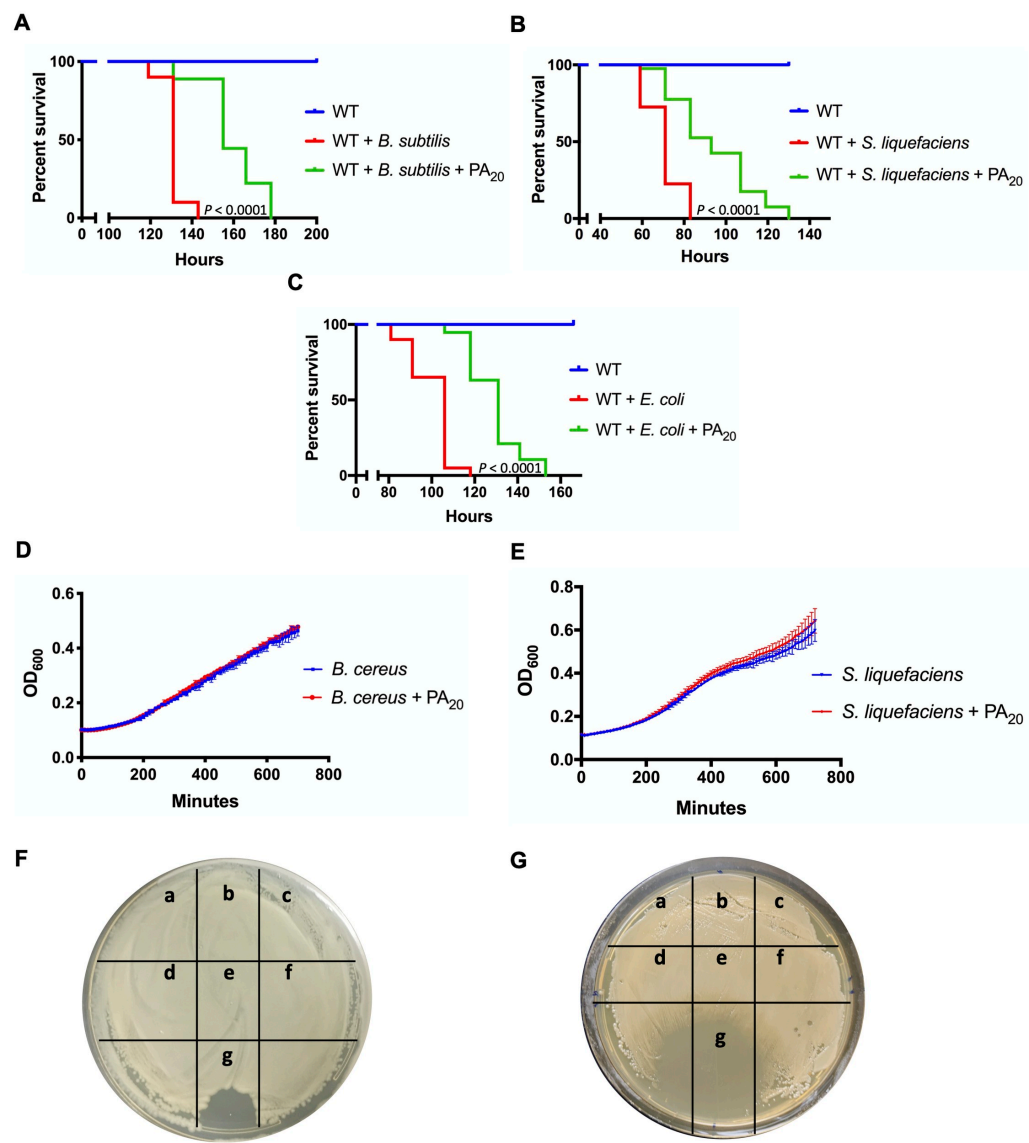
PA_20_ protection is broad-spectrum and insect-directed. (A-C) The effect of PA_20_ on the sensitivity of flies to other bacteria. WT flies were infected with *B*. *subtilis* (A), *S*. *liquefaciens* (B), and *E*. *coli* (C) without or with PA_20_. *P* as in Fig 1. (D-G) PA_20_ does not alter the growth of *B*. *cereus* and *S*. *liquefaciens*. (D-E) The ability of PA_20_ to affect the rate of *B*. *cereus* (D) and *S*. *liquefaciens* (E) growth in LB and TSB, respectively. The bacterial growth was measured in a liquid medium in a 96-well plate with and without 1 μg/mL of PA_20_. Each data point shown indicates the mean ± SD value obtained in triplicate assays done in a representative experiment. (F-G) Agar diffusion susceptibility assay of *B*. *cereus* (F) and *S*. *liquefaciens* (G). LB and TSB plates were treated with 1 μL of toxins right after the spreading of *B*. *cereus* and *S*. *liquefaciens* cultures, respectively, and left to incubate overnight. Spots below a-g contain a 1 μL spot of: 1 μg/mL PA_83_ (a), PA_63_ (b), PA_20_ (c), LF (d), EF (e), PBS (f), and 10 mM levofloxacin (g).

The effects of PA_20_ extend beyond bacteria of the *B*. *cereus* group, as it enhanced the survival of flies exposed to related *B*. *subtilis* and unrelated Gram-negative *Serratia liquefaciens* and *Escherichia coli*. PA_20_ extended the median survival time of *B*. *subtilis* infected flies by 24 hours (*P* < 0.0001), *S*. *liquefaciens* infected flies by 22 hours (*P* < 0.0001), and *E*. *coli* infected flies by 25 hours (*P* < 0.0001) although some flies were protected by as long as 35–47 hours (Fig 4A–4C). As with the wild-type flies infected with *B*. *cereus* (S2 Fig), we observed that a group of flies of various ages infected with *S*. *liquefaciens* was protected by PA_83_ (delay of the median survival time by 152 hours, *P* < 0.0001) (S4 Fig). To test whether PA_20_ exerted its protective effects by suppressing the growth of bacteria, we measured the growth rates of bacteria in the presence or the absence of PA_20_. PA_20_ did not affect the growth of *B*. *cereus* and *S*. *liquefaciens* in liquid culture (Fig 4D and 4E) or on solid media (Fig 4F and 4G), suggesting that PA_20_ reduces the sensitivity of flies to insecticidal bacteria through an insect-oriented mechanism.

### PGRP-SA binds to PA_20_ and is necessary for PA_20_-mediated protection

**Fig 5:**
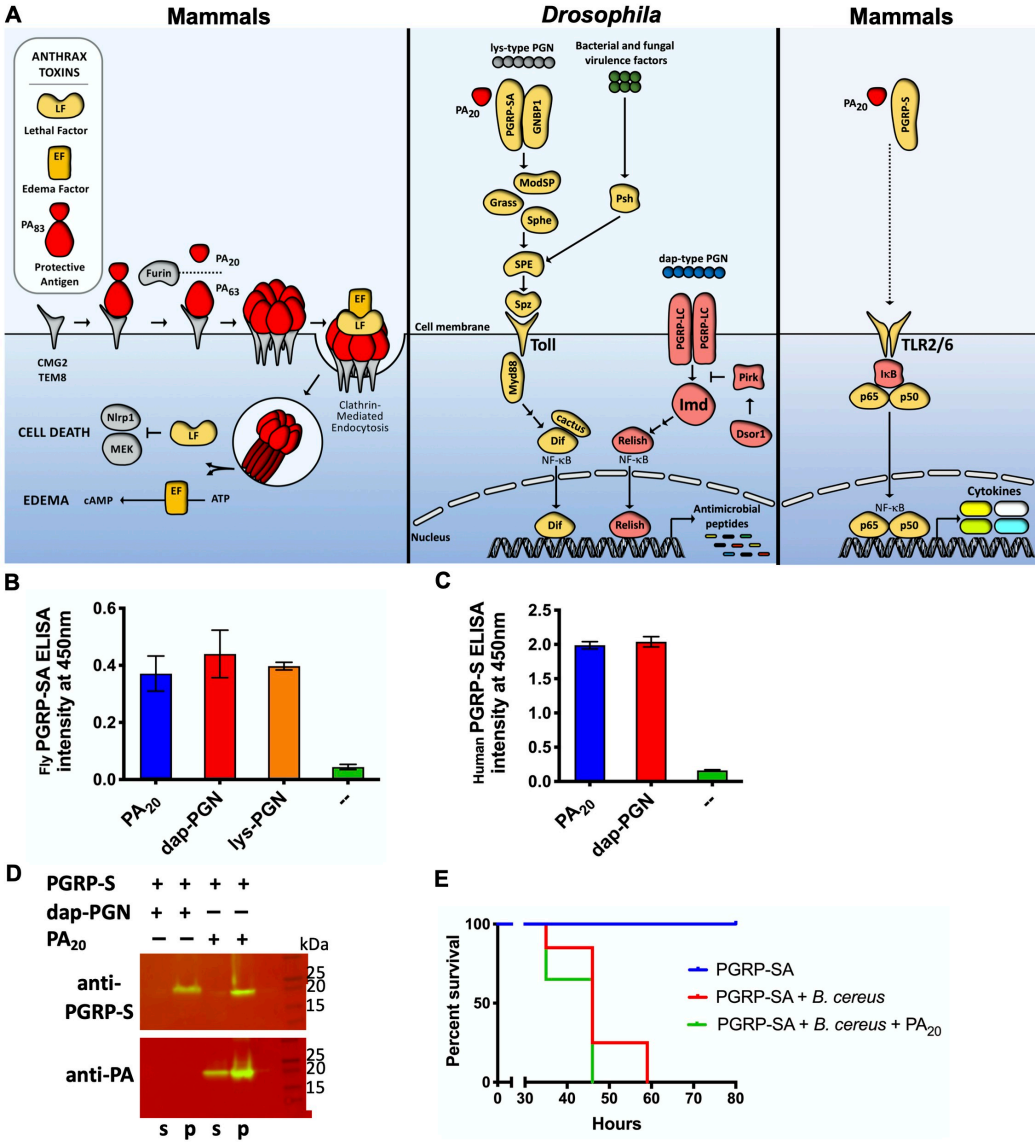
Fly PGRP-SA interacts with PA_20_ and is necessary for PA_20_-mediated protection. (A) A model of PA_20_-mediated phenomenon. During mammalian anthrax infection (left panel), anthrax PA_83_ binds to host CMG2 and TEM8 cell surface receptors. PA_83_ is then cleaved by cell surface mammalian furin proteases, yielding PA_20_ and PA_63_, with the latter remaining attached to the host cell receptor. PA_63_ then multimerizes and binds to the circulating LF or EF molecules. This PA pre-pore is endocytosed with the help of clathrin. Upon entry into the cell, the PA pre-pore becomes the PA-pore. This change allows LF and EF to escape into the cell cytosol. Once in the cytosol, EF functions as an adenyl cyclase resulting in edema. Conversely, LF cleaves MAPKK and Nlrp1, causing caspase-mediated pyroptosis. Upon contact with anthrax infected animals or their carcasses, scavenging insects are likely exposed to PA_20_. PA_20_, once ingested by *Drosophila*, may interact with the Toll pathway to reduce the sensitivity of flies to bacterial challenge (center panel). Two pathways trigger the expression of antimicrobial peptides in *Drosophila*: the Toll and Imd pathways. During the bacterial challenge, Toll is activated by bacterial lys-type peptidoglycan (PGN) by binding to the PGRP-SA/GNBP1 heterodimer and undergoing enzymatic modification. This results in Toll activation by SPZ ligand binding. Concurrently or separately, Toll is also known to be activated by the circulating virulence factor detection protein, Psh, which also works through SPZ to induce Toll activation. The Imd pathway responds primarily to bacterial dap-type peptidoglycans such as that found in *B*. *cereus* and *S*. *liquefaciens*. Both Toll and Imd ultimately activate NF-κB transcription factors, which induce the transcription of AMPs that act in a broad-spectrum manner. PA_20_, once ingested by *Drosophila*, works through Toll and likely PGRP-SA to reduce the sensitivity of flies to bacterial challenge. The effect of PA_20_ on the Toll/NF-κB pathway may also occur on the homologous mammalian pathway (right panel). The vaccination with PA_83_ and human cell exposures to PA_83_ or PA_20_ are known to induce the Toll-like receptors-mediated activation of NF-κB and inflammatory cytokines, such as Interleukins. (B-C) ELISA assays showing the interaction between *D*. *melanogaster* PGRP-SA (B) or human PGRP-S (C) with PA_20_ or peptidoglycans (PGN). PGRP-SA or PGRP-S are added to wells with immobilized PA_20_ or PGNs. Anti-T7 antibody is used to detect T7-tagged PGRP-SA, and anti-PGRP-S antibody is used to detect PGRP-S. (D) PGRP-S pull-down assay. PGRP-S binds to and co-precipitates with the partially soluble PA_20_ or the insoluble *B*. *subtilis* peptidoglycan (PGN). The supernatant (s) or pellet (p) samples from the pull-down assay were analyzed by the Western blot using anti-PGRP-S (top) or anti-PA (bottom) polyclonal antibodies. (E) The effect of PA_20_ on PGRP-SA mutant flies. *Drosophila* mutants were challenged with *B*. *cereus* with or without PA_20_.

Lys-type peptidoglycans are recognized by a circulating extracellular heterodimer receptor consisting of peptidoglycan-recognition protein-SA (PGRP-SA) and GNBP1 [19, 51] (Fig 5A). This peptidoglycan binding triggers a proteolytic cascade that includes ModSP, Grass, Sphe, Psh, and SPE [18]. Ultimately, SPE cleaves pro-Spätzle to release the mature Toll ligand, Spätzle (Spz), and trigger the subsequent intracellular Toll signaling leading to the degradation of the inhibitor of κB, cactus, the activation of NF-κB (Dif), and the transcription of AMPs.

The five amino acid consensus sequence of lys-type peptides recognized by PGRP-SA is AQKA/SA/S [52]. The PA_20_ portion of the 1β_13_−1α_1_ loop contains a similar sequence: 186-KQKSS-190. To test whether the protective capacity of PA_20_ might be due to its ability to bind to PGRP-SA (Fig 5A), we used purified recombinant *D*. *melanogaster* PGRP-SA and its human orthologue PGRP-S, as they share 60% sequence homology and the peptidoglycan-binding site is closely conserved between the two proteins [53, 54]. Moreover, just like PGRP-SA, PGRP-S binds to lys- and dap-type peptidoglycans (dap is a derivative of lys) [54]. We performed an ELISA, where PRGP-SA or PGRP-S was added to plates coated with PA_20_ or peptidoglycans. PRGP-SA (Fig 5B) and PGRP-S (Fig 5C) bound PA_20_ and peptidoglycans and were detected with anti-PGRP antibodies.

To validate this observation, we analyzed the ability of PGRP-S to bind to PA_20_ in a co-precipitation assay, which relied on the observation that dap-type peptidoglycan and PA_20_ are partially insoluble. We incubated PGRP-S with either peptidoglycan or PA_20_, followed by centrifugation and washing of pellets. Western blot analysis demonstrated that PGRP-S bound to PA_20_ and peptidoglycan, and thus, was pulled down into pellets (Fig 5D). Moreover, PA_20_ did not affect the sensitivity of PGRP-SA loss-of-function mutant flies to *B*. *cereus*, showing that this gene is necessary for the PA_20_-mediated phenomenon (Fig 5E). Collectively, these results suggest that PA_20_ may exert its protective effects in *Drosophila* by binding to PGRP-SA (Fig 5A).

### The Toll pathway is necessary, and the Imd pathway is sufficient for PA_20_-mediated protection

**Fig 6:**
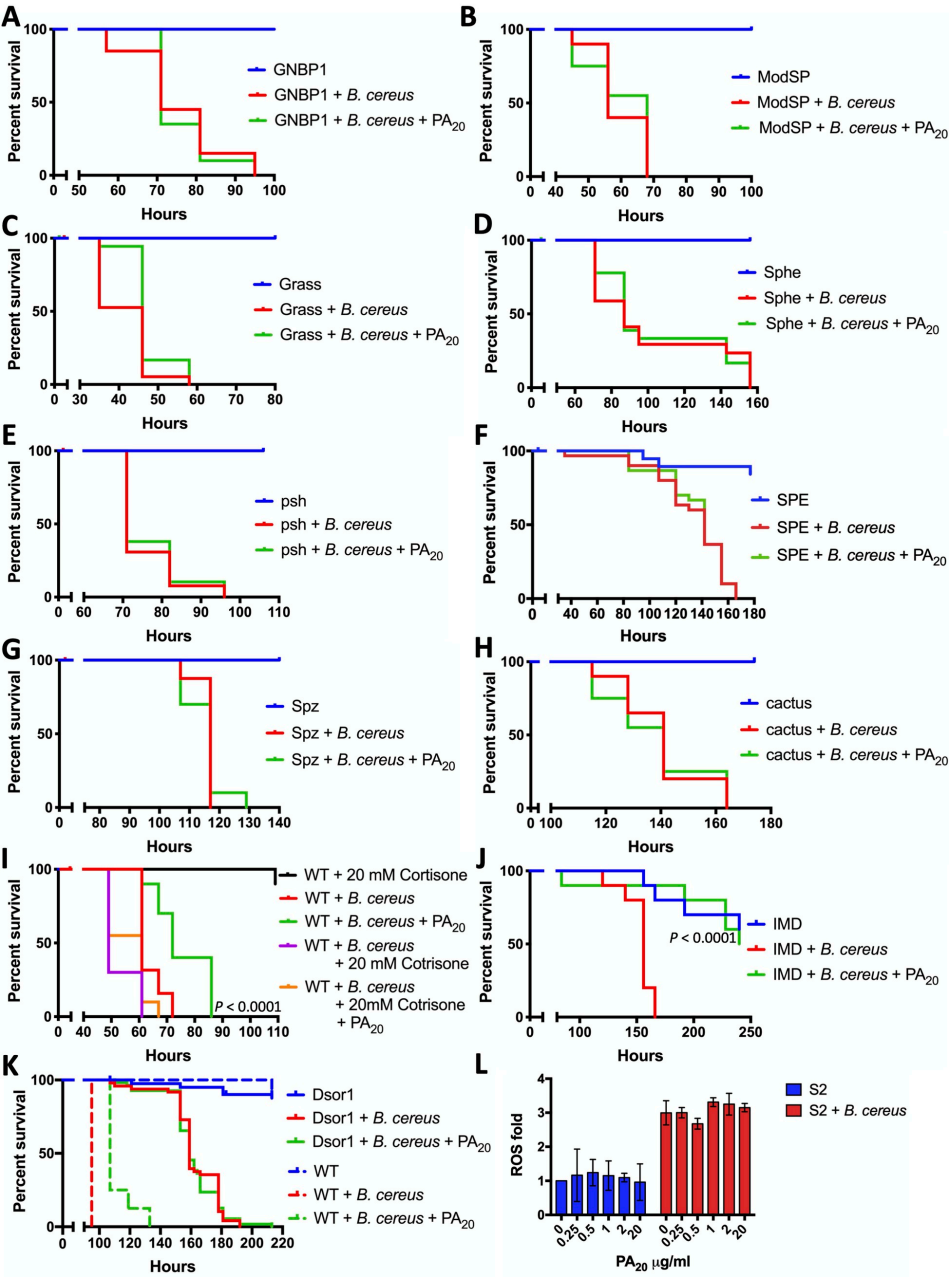
The effect of the Toll and Imd pathways and phagosomal ROS on PA_20_-mediated protection. The effect of PA_20_ on the Toll pathway mutants. Gram-Negative Bacteria Binding Protein 1 (GNBP1) (A), Modular Serine Protease (ModSP) (B), Gram-positive Specific Serine protease (Grass) (C), Spheroide (Sphe) (D), Persephone (Psh) (E), Spätzle Processing Enzyme (SPE) (F), Spätzle (Spz) (G), and cactus (H). *Drosophila* mutants were challenged with *B*. *cereus* with or without PA_20_. WT (I) and (J) mutant flies were infected with *B*. *cereus*, without or with 1 μg/mL PA_20_ or 20 mM Cortisone acetate. Female WT and Dsor1 (K) mutant flies were tested with and without PA_20_. *P* as in Fig 1. (L) Measuring reactive oxygen species (ROS) consisting of hydrogen peroxide, peroxynitrite, hydroxyl radicals, nitric oxide, and peroxy radicals in *Drosophila* macrophage-like plasmatocytes S2 cells in the absence and the presence of PA_20_. Radicals were measured using ROS-ID Total ROS/Superoxide Detection Kit (Enzo Life Sciences). Radicals produced by S2 cells were measured in the absence or the presence of *B*. *cereus* and various concentrations of PA_20_ (0.25 to 20 μg/mL).

We investigated whether the Toll pathway, the Imd pathway, and the phagosomal ROS production are involved in PA_20_-mediated protection of infected flies. Toll pathway GNBP1, ModSP, Grass, Sphe, Psh, SPE, Spz, and cactus loss-of-function mutants were infected with *B*. *cereus* in the absence and the presence of PA_20_ (Fig 6A–6H). PA_20_ did not protect any of the Toll-pathway mutants infected with *B*. *cereus*. We confirmed the inability of PA_20_ to protect SPE and Spz mutants challenged with *S*. *liquefaciens* (S5A and S5B Fig). We tested the ability of a known immunosuppressive inhibitor of NF-κB, cortisone acetate [55], to sensitize flies to *B*. *cereus* and suppress PA_20_-mediated protection. The addition of a minimal immunosuppressive concentration of cortisone acetate (20 mM) to the feeding medium increased the sensitivity of *Drosophila* to *B*. *cereus* and negated the protective effects of PA_20_ (Fig 6I and S6 Fig) without affecting the growth of *B*. *cereus* (S3 Fig).

We evaluated whether Imd is necessary for PA_20_-mediated protection by testing the ability of PA_20_ to reduce the sensitivity of Imd-deficient mutant flies to bacterial infections. The results revealed that PA_20_ was able to delay *B*. *cereus*-induced (Fig 6J) and *S*. *liquefaciens*-induced (S5C Fig) mortality of Imd mutants.

We then evaluated whether the activity of the Imd pathway is sufficient for PA_20_-mediated protection. One of *D*. *melanogaster*’s mitogen-activated protein kinase kinases, Dsor1, acts as a suppressor of the Imd pathway, and the downregulation of Dsor1 mimics the induction of the Imd pathway by microbes, even in the absence of an immune challenge [56]. Dsor1 blocks the Imd pathway by activating Pirk, which, in turn, interacts directly with PGRP-LC and PGRP-LE and disrupts their interaction with Imd [57] (Fig 5A). We tested the ability of PA_20_ to reduce the sensitivity of Dsor1 mutant flies to *B*. *cereus* infection. We observed that these mutant flies are less sensitive to *B*. *cereus* infection in the absence of PA_20_, and that the addition of PA_20_ has no effect on Dsor1 mutant fly sensitivity (Fig 6K). We confirmed the inability of PA_83_ and PA_20_ to protect Dsor1 mutant flies infected with *S*. *liquefaciens* (S5D and S5E Fig). Collectively, these data demonstrate that while Imd is not necessary for the PA_20_-mediated phenomenon, the constitutive activity of the Imd/NF-κB pathway in Dsor1 mutant flies is sufficient to negate the protective effects of PA_20_.

We examined whether PA_20_ induced the production of reactive oxygen species (ROS) in *Drosophila* macrophage-like plasmatocytes, S2 cells. We measured the total ROS and separately superoxide radicals produced by S2 in the absence or the presence of *B*. *cereus* and various concentrations of PA_20_ (0.25 to 20 μg/mL) (Fig 6L and S7 Fig). Although *B*. *cereus* increased the abundance of ROS in S2, the addition of PA_20_ did not further alter the levels of these species. Our overall results demonstrate that the Toll pathway is necessary and the Imd pathway is sufficient but not necessary for the PA_20_-mediated phenomenon, and that PA_20_ does not affect phagosomal ROS production.

### Toxins of other bacterial pathogens may help flies resist anthrax

**Fig 7:**
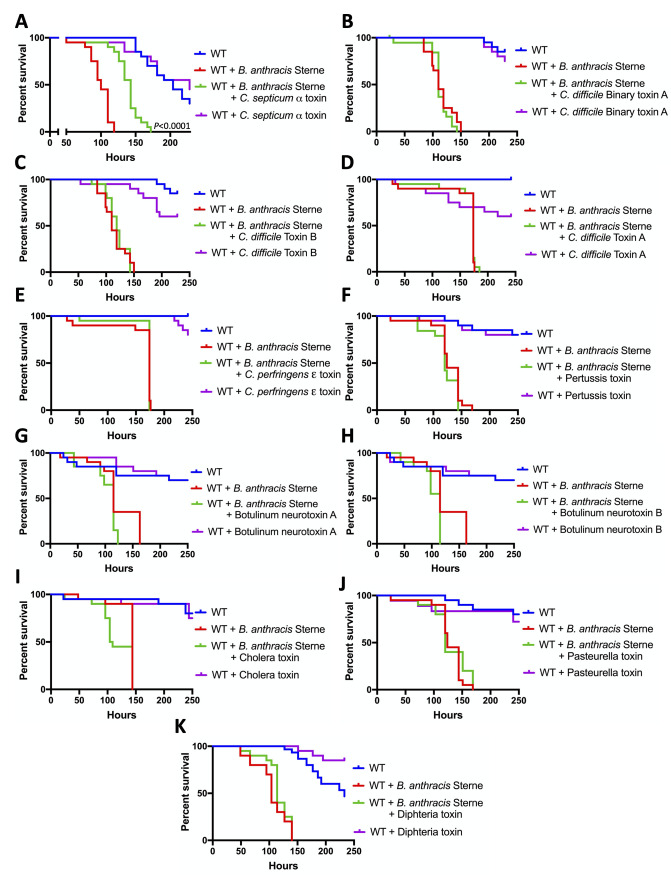
The effect of various bacterial toxins on the sensitivity of *Drosophila* to *Bacillus anthracis* Sterne strain. Male Oregon-R wild type (WT) flies were orally challenged with vegetative *B*. *anthracis* Sterne strain in the absence or presence of bacterial toxins: *Clostridium septicum* alpha toxin (A), *C*. *difficile* binary toxin subunit A (B) and toxins B (C) and A (D), *C*. *perfringens* epsilon toxin (E), pertussis toxin (F), botulinum neurotoxins A (G) and B (H) (heavy chains), cholera toxin (I), Pasteurella toxin (J), and diphtheria toxin (K). All toxins were tested orally at 1 μg/ml in a sucrose solution. The effect of each toxin on fly survival was tested in the absence of bacteria as a control. *P* as in Fig 1.

During the infection, anthrax-causing bacilli may co-exist with other environmental and commensal pathogenic agents. Consequently, anthrax-exposed insects can also be co-exposed to toxins of other bacteria. We investigated whether exotoxins of eight pathogenic bacteria affect the sensitivity of flies to *B*. *anthracis* Sterne strain (Fig 7A–7K). We observed that *Clostridium septicum* alpha toxin reduces the sensitivity of flies to *B*. *anthracis* Sterne strain infection (delay of the median survival time by 43 hours, *P* < 0.0001) (Fig 7A), demonstrating that toxins from other microorganisms may help flies resist anthrax.

## Discussion

While biting and non-biting flies have been known to be capable of transmitting *B*. *anthracis* since the 19th century [58–60], the mechanism of insect-mediated anthrax transmission is unknown. Recent studies emphasized that anthrax and anthrax-containing insects pose a significant ecological threat in West African ecosystems [2–4, 7–11]. When flies feed on anthrax-infected or deceased mammals, they invariably ingest both *B*. *anthracis* and circulating anthrax toxin components, including PA_20_ [37]. *B*. *anthracis* is known to remain in the insect’s gut for an extended time or as part of the insect microbiota for life [7]. While the potential benefit of this co-existence has not been studied, we propose a mechanism by which the vector-borne pathogen may benefit. *Bacillus* species, which naturally reside in the soil, are relatively nonmotile and benefit from a suitable vector to assist bacterial distribution. The reduced insect sensitivity would benefit mammalian pathogens by allowing microbes to further their reach.

Our study exposed insects to vegetative *B*. *anthracis* for days, while in previous studies blow flies, horn flies, house flies, mosquitoes were found to be insensitive to this pathogen after short exposures for several hours [11, 15, 17, 39], possibly explaining the difference in the insect sensitivity between studies. Moreover, we demonstrate that *Drosophila* and *Culex* are insensitive to *B*. *anthracis* spores, which is similar to previous observations that *B*. *anthracis* spores are not lethal to flies, mosquitoes [11, 15, 17, 39], and nematodes [61, 62]. Our results revealed that flies and mosquitoes respond differently to spores. Spores from either the Sterne or ΔSterne strains extended the longevity of *Drosophila*, with toxin-containing Sterne strain extending the life longer than the ΔSterne strain, whereas the longevity of mosquitoes was not affected by spores of either strain (Fig 3). It is possible that in our experimental conditions, uninfected flies can succumb to environmental microorganisms after 10–14 days, and the oral exposure to *B*. *anthracis* spores extended the longevity of flies by stimulating their innate immunity. Since it was previously shown that PA is detected on the surface of *B*. *anthracis* spores [63, 64], we hypothesize that the presence of this surface PA proteins could further activate the innate immunity of flies and extend their longevity, as we observed in this study. The reason for the lack of the extension of longevity in mosquitoes could be that the cause of death of mosquitoes may be different from flies or because spores do not affect the immunity the same way they do in flies. Moreover, our results revealed that unlike *Drosophila*, mosquitoes sensitivity was similar to Sterne and ΔSterne strains. We conjecture this could occur due to mosquitoes’ comparatively superior immune system, but this observed difference was not further explored. Future studies should investigate the toxin-dependent and -independent interaction of *B*. *anthracis* with flies and mosquitoes.

This work describes a new function of PA_20_, the domain that previously has only been known to prevent self-assembly of PA_83_ in solution. PA_20_ has also been known to contribute to the adaptive immunogenicity of PA_83_ in mammals, as it contains at least three immunogenic epitopes [32]. One such PA_20_ epitope is the 1β_13_−1α_1_ loop shown to affect insect and plant innate immunity in this and previous studies [48]. The results described above led us to propose a novel mechanism by which PA_20_ reduces the sensitivity of flies to insecticidal bacterial pathogens by utilizing *Drosophila*’s Toll pathway (Fig 5A). Both *Bacillus* and *Serratia* species of bacteria are known to possess dap-type peptidoglycans and thus are known inducers of the Imd pathway [52]. Toll pathway is required for PA_20_ function, and PA_20_ may directly interact with *Drosophila* PGRP-SA to activate Toll, while *Bacillus* bacteria activate Imd, which would result in the transcription of AMPs [23]. Independently and together, Toll and Imd pathways result in the expression of AMPs that are known to be broad-spectrum in their effects and may possess the ability to act additively and/or synergistically [23, 65]. PA_20_ may be providing insects with an added benefit, resulting in the extension in lifespan during the bacterial challenge and allowing pathogens to further their reach. Alternatively, as dap is a structural derivative of lys, and since both Toll and Imd pathways can detect lys- and dap-peptidoglycan [21, 54], it is possible that PA_20_ may bind to Imd-activating PGRP-LC and PGRP-LE, thus leading to the activation of both Toll and Imd pathways. Although we show that the Imd pathway is not necessary, its activity is sufficient to negate the protective effects of PA_20_.

*Drosophila* GNBP-1 and PGRP-SA form a functional heterodimer. Fly PGRP-SA binds predominantly to lys-type peptidoglycan, but also binds to dap-type peptidoglycan [19]. In contrast, GNBP-1 only binds to lys-type peptidoglycan [19]. GNBP-1 is known to hydrolyze Gram-positive peptidoglycan, while PGRP-SA binds peptidoglycan fragments (muropeptides). GNBP-1 presents a hydrolyzed form of peptidoglycan for sensing by PGRP-SA, and this tripartite interaction between these proteins and peptidoglycan fragments is essential for downstream signaling. Future studies should establish whether GNBP-1 and PGRP-SA form a tripartite interaction and whether this leads to the activation of the downstream Toll pathway.

In *Drosophila*, at least seven AMPs (plus their isoforms) have been described: Diptericins (DptA and DptB), Cecropins (CecA1, CecA2, CecB, CecC, and Cecψ1), Drosocin (Dro), Attacins (AttA, AttB, AttC, and AttD), Drosomycins (Drs, Dro2, Dro3, Dro4, Dro5, Dro5, and Drsl1), Metchnikowin (Mtk), and Defensin (Def), with many of them showing broad-spectrum antimicrobial activity against Gram-negative and Gram-positive bacteria [23–25]. The expression of many of them has been shown to be regulated at the transcriptional level by both the Toll and the Imd pathways and by both Gram-negative and Gram-positive bacteria, where the time of AMPs expression post-infection varies for each peptide [23–25]. Moreover, the tissue expression of AMPs follows a complex pattern that is specific for each peptide, where individual AMPs can be expressed in the digestive tract, salivary glands, labellar glands, respiratory tract, seminal receptacle, spermatheca, calyx, and oviduct [24]. Future studies should determine: i) the identity of AMPs activated in response to the exposure of flies to PA_20_, ii) the tissues where AMPs are expressed in response to PA_20_, and iii) the timing when AMPs are expressed in response to PA_20_.

In addition to AMP and hemocyte responses, fly intestinal epithelia exposed to pore-forming toxins undergo an evolutionarily conserved process of thinning (purging) followed by the rapid recovery of their initial thickness [66]. We argue that while PA is a pore-forming toxin component, the effect of PA_20_ is independent of pore formation, as it lacks PA domain 3, which is located within PA_63_ and is necessary for PA_63_-multimerization [67]. We propose that the mechanism of action by which the pore-forming *Clostridium septicum* alpha toxin provides flies protection from *Bacillus anthracis* Sterne strain infection is by inducing the purging of fly intestinal epithelia, as described by Lee *et al*. [66]. Future studies should explore this hypothesis.

The effect of PA_20_ on the Toll/NF-κB pathway observed in this study is consistent with the impact PA_83_ and PA_20_ have on the homologous mammalian pathway (Fig 5A, right panel). PA_83_ is the central antigen in the FDA-approved anthrax vaccine, BioThrax [68]. The vaccination with PA_83_ [38] and human cell exposures to PA_83_ [36] or PA_20_ [37] lead to the Toll-like receptors-dependent activation of NF-κB and a subsequent upregulation in the expression of pro-inflammatory cytokines, especially Interleukin-6 (IL-6), IL-6 receptor, IL-1β, and Tumor necrosis factor alpha (TNF-α). Thus, in addition to an established paradigm of PA_83_ activating adaptive immunity in mammals, this toxin component may also affect innate immunity. BioThrax is approved for use as both a pre-exposure vaccination and post-exposure prophylaxis [69, 70], which is consistent with the potential that its efficacy is partly based on an unrecognized effect on the human innate immune system, as presented here.

While our study may help understand the mechanism through which insects tolerate anthrax, resulting in their greater opportunity to transmit the anthrax-causing bacteria, it further suggests that PA_20_ could be used beneficially in agriculture. Since fruit flies and mosquitoes are known to act as plant pollinators [71, 72], PA_20_ should be further evaluated for its ability to reduce the sensitivity of pollinating insects to bacterial pathogens, such as *Serratia* and *Bacillus* species [73–75].

## Materials and methods

### Chemicals and reagents

Anthrax toxin components used: PA_83_, PA_63_, and LF were a kind gift from Kenneth Bradley (University of California, Los Angeles). Toxins (product numbers) purchased from List Biological Laboratories were: EF (178A), diphtheria toxin (150), *Clostridium septicum* alpha toxin (116L), *C*. *perfringens* epsilon toxin (126A), *C*. *difficile* toxins A (152C), B (155B), and binary toxin subunit A(157A), Pasteurella toxin (156), pertussis toxin (181), botulinum neurotoxins A (612A) and B (622A) (heavy chains), and cholera toxin (100B). PA_20_ was recombinantly expressed using the methods described below. Purified recombinant T7-tagged *D*. *melanogaster* PGRP-SA protein was a kind gift from Sérgio Raposo Filipe (Universidade Nova de Lisboa). Chemical/reagents (source, catalog #) are: cortisone acetate (TCI, C0389), human recombinant PGRP-S (also called PGLYRP1) (BioVendor, RD172316100), polyclonal mouse anti-PGRP-S antibody (Abnova, H00008993-B01P), monoclonal mouse Anti-T7 tag antibody (Sigma-Aldrich, T8823), peptidoglycan from *B*. *subtilis* and *Staphylococcus aureus* (Sigma-Aldrich, 69554 and 77140, respectively), polyclonal goat anti-PA antibodies (List Biological Laboratories, 771B), rabbit anti-goat-HRP secondary antibody (Invitrogen, 81–1620), and goat anti-mouse-HRP secondary antibody (Bio-Rad, 172–1011). PA_83_ and PA_20_ 1β_13_−1α_1_ loop peptides were custom synthesized with purity higher than 95% and lyophilized by LifeTein using the method described below and resuspended in PBS at 1 mg/mL.

PA_20_ peptides were synthesized by LifeTein on ChemMatrix Rink Amide resin, using standard Fmoc synthesis protocol with DIC/Cl-HOBt coupling, on an APEX 396 automatic synthesizer. The resin was swollen in N,N-Dimethylformamide (DMF) for 30 minutes, treated with 20 v% Piperidine-DMF for 8 minutes to remove the Fmoc protecting group, at 50°C, and washed with DMF for three times. For the coupling reaction, the resin was added with Fmoc-protected amino acid, Cl-HOBt, DIC, and NMP. The mixture was vortexed for 20 minutes at 50°C. Afterward, the resin was washed with DMF once. The cycle of deprotection and coupling steps was repeated until the last amino acid residue was assembled. After the final Fmoc protecting group was removed, the resin was treated with 20 v% acetic Anhydride-NMP for 20 minutes. The resin was then washed with DMF, DCM, and dried with air. The peptides were cleaved using a TFA cocktail (95 v% TFA, 2.5 v% water, and 2.5 v% TIS) for three hours. Crude peptides were precipitated by adding ice-chilled anhydrous ethyl ether, washed with anhydrous ethyl ether three times, and dried in vacuo. The crude peptides were HPLC purified.

### *Drosophila* rearing

*D*. *melanogaster* fly strains were maintained at 25 ˚C with 12-hour light/dark cycles and fed standard cornmeal-molasses-agar fly medium with yeast flakes. Wild-type (WT) experiments were conducted with Oregon-R *Drosophila* (Bloomington *Drosophila* Stock Center (BDSC) stock # 5) aged 4–5 days. Some experiments were performed with wild-type flies of various ages. All mutant flies were of various ages at the time of experimentation. Mutant fly strains used (and their BDSC stock #) are: Imd (55711), cactus (34501), SPE (33926), Spz (3115), GNBP1 (18150), Sphe (29227), Psh (52877), PGRP-SA (58589), ModSP (55717), Grass (67099) and Dsor1 (5545). For Dsor1 experiments, only female flies were tested because male flies are short-lived post-eclosure (S8 Fig).

### Mosquito rearing

*Culex quinquefasciatus* mosquitoes were obtained from a colony maintained by Benzon Research (Carlisle, PA, USA). *Culex* larvae were fed daily with 0.025 g/L of a 3:1 mixture of bovine liver powder and brewer’s yeast (Benzon Research). Mosquito larvae were maintained at 200 larvae per 1 liter of nanopore water in 35.6 cm x 27.9 cm x 8.3 cm plastic trays. Mosquitoes were reared and maintained at 28°C, 12:12 hours light:dark diurnal cycle at 80% relative humidity in 30 × 30 × 30-cm cages. Mosquitoes were provided with 10% sucrose *ad libitum* for maintenance. For experiments, mosquitoes aged 3–4 days were used.

### Bacterial strains and spore preparation

*B*. *cereus* (ATCC 10987), *B*. *subtilis* (strain 168), *Escherichia coli* (K-12 MDS42), and *S*. *liquefaciens* (ATCC 27592) were used as the infective agents for *Drosophila* survival assays. *B*. *cereus* and *B*. *subtilis* were incubated on Lysogeny Broth (LB) at 37°C while *S*. *liquefaciens* was grown on Tryptic Soy Broth at 30°C. Overnight cultures were made in the respective media and temperatures at 180 rpm for 16–18 hours.

Strains of *B*. *anthracis* used included the toxigenic Sterne and toxin-negative ΔSterne strains. Spores were prepared and purified from solid agar (NBY-Mn) cultures of the strains and purified as described [76, 77]. Medium components were from BD-Difco, and the NBY-Mn medium was composed of nutrient broth (8 g/L) (BD 23400); yeast extract (3 g/L) (BD 244020); MnSO4·H2O (25 mg/L) (Sigma-Aldrich, M7634); and agar (15 g/L) (Aldon Corp, AA0075). Spores were used only if >95% were refractile (ungerminated) as determined by phase microscopy and heat-resistance. Spore preparation included two centrifugations in density gradient medium (58 mL Hypaque-76™, Nycomed into 42 mL WFI) accompanied by extensive washing in sterile water for injection (WFI). The spores were activated by heating at 65°C for 30 min just before use in assays [76].

### Oral feeding survival assay

Flies or mosquitoes were infected according to the bacterial intestinal infection methods previously employed by Nehme *et al*. [78] with minor modifications. To prepare the *Drosophila* or *Culex* vials for infection, three pre-cut extra-thick Whatman blotting papers (Bio-Rad Laboratories, 1703965) were stacked at the bottom of either 25x95 mm diameter polystyrene *Drosophila* vials or 55x55x102 mm specimen bottles for fly and mosquito infections, respectively, following by capping with a foam plug. Overnight bacterial cultures were centrifuged, and the bacterial pellets were resuspended in either a 50 mM or 10% sucrose solution for fly or mosquito experiments, respectively.

Insects were exposed orally to anthrax toxin components, domains, or peptides, each at a concentration of 1 μg/mL, which falls within the known 0.01–100 μg/mL range of the concentration of plasma-circulating toxin components in infected mammals during the late stage of anthrax infection [79–88]. All other bacterial toxins were tested at the same concentration.

Flies and mosquitoes were exposed orally to approximately 2.6 X 10^9^ cells/mL, as previous studies have shown that the concentration of plasma-circulating bacteria in infected mammals during the late stage of anthrax infection is 10^9^−10^10^ cells/mL [80, 81, 88]. Oral exposure was chosen because it represents a route by which flies are exposed to anthrax toxin components and bacilli in a natural environment [18]. All experiments were performed with 4–5 day old (post-eclosion) wild type male flies and mosquitoes to reduce any potential variability in sensitivity to bacterial infections due to confounding gender and age differences.

A final optical density at 600 nm (OD_600_) was 3.3 for *Bacilli* species and *E*. *coli* (2.6 X 10^9^ cells/mL), as well as OD_600_ of 4.6 for *S*. *liquefaciens* (3.7 X 10^9^ cells/mL). Depending on the experimental condition, toxin components and cortisone acetate were also added to bacterial sucrose solutions before adding to *Drosophila* vials and mosquito bottles. Finally, 2.5 or 10 mL of the respective solution was pipetted onto the Whatman paper in each *Drosophila* vial or mosquito bottle. Flies and mosquitoes were anesthetized using CO_2_, separated by gender, and placed ten at a time into their respective vials or bottles and incubated at 30°C. The survival of insects was recorded a minimum of twice per day.

### PA domains expression and purification

A gene strand containing a *pelB* leader sequence, the amino-terminal of PA_20_ (residues 30–192), a 6-His tag, and 20 bp vector overlaps at the 5’ and 3’ end was ordered from Eurofins Genomics. PA_83_ amino acids 1–29 were not included in the expression, because they form a signal peptide that is cleaved off of the mature protein [89]. Digested pSX2 expression vector (Scarab Genomics) and the *pelB*-PA_20_-6His gene strand were assembled using Gibson assembly.

PA domains 2, 3, and 4 fusion proteins were designed to include the PA domain, a C terminal InaD PDZ domain (amino acids 2–98, GenBank accession no. 1IHJ_A, cys53ala mutation), a C-terminal 6xHis tag, and 3’ and 5’ overlaps for insertion into the pSX2. The gene strands were cloned into KpnI and SacI-digested pSX2 using the NEBuilder HiFi DNA Assembly Cloning Kit (New England Biolabs, E5520S).

The sequences for the His-tagged PA_D1_ and PA_D1’_-InaD fusion proteins were PCR amplified with vector overlaps to IPTG-inducible expression vector containing maltose-binding protein (MBP) with a C-terminal PreScission protease cleavage site (pET His6 MBP prescission LIC cloning vector (HMPKS, plasmid #29721)). The vector was digested using XhoI and SspI, and the PCR-amplified inserts were cloned in using the NEBuilder HiFi DNA Assembly Cloning Kit.

All constructed plasmids were transformed into chemically competent *Escherichia coli* DH5α cells for plasmid preparations. Purified PA_20_, PA_D2_, PA_D3_, and PA_D4_ plasmids were then transformed into MDS42 *E*. *coli* for expression. Purified PA_D1_ and PA_D1’_ plasmids were transformed into chemically-competent T7 Express *E*. *coli* (New England Biolabs, C2566I) for protein expression. His-tagged PA_20_, PA_D1_, and PA_D1’_ were purified using Ni-NTA resin (Thermo Fisher Scientific, 88221), and PA_D2_, PA_D3_, and PA_D4_ were purified on HisTrap HP columns (GE Healthcare, GE29-0510-21).

MBP was cleaved from the MBP- PA_D1_, and -PA_D1’_ fusion proteins in solution using the Pierce HRV 3C Protease Kit (Thermo Scientific, 88946). The HRV3C protease was removed using Pierce Glutathione Agarose (Thermo Scientific, 16100) and Pierce Centrifuge Columns (Thermo Scientific, 89898). MBP was removed using amylose resin (New England Biolabs, E8021S), followed by dialysis and concentration of proteins using Vivaspin Centrifugal Concentrators (Sartorius, VS0101). Protein concentration was determined using the Pierce BCA Protein Assay Kit with bovine serum albumin as a standard (Thermo Scientific, 23225).

All PA domains were analyzed via SDS-PAGE and anti-PA_83_ Western blotting to confirm the molecular weight using polyclonal goat anti-PA_83_ and the secondary rabbit anti-goat IgG antibodies. Chemiluminescence of bands and their relative intensities were revealed using Azure c500 (Azure Biosystems, Dublin, CA).

### Bacterial growth assays

For the agar-containing media assays, the OD_600_ of overnight cultures of bacteria was measured. The OD_600_ values were converted to cells/mL according to McFarland’s scale [90]. 6 X 10^8^ bacterial cells were added to solid media on 25 cm Petri dishes. One μL of toxin component or cortisone acetate was then pipetted onto the plate surface. The plates were then incubated for 24 hours at the temperatures appropriate for each bacterium. One μL of 10 mM levofloxacin (Cayman Chemical, 20382) or phosphate-buffered saline (PBS) (Corning, 21-040-CV) were included as controls.

In the liquid media experiments, the bacterial overnight culture was resuspended in a new liquid bacterial medium to OD_600_ of 0.1. One hundred μL of the bacterial solution were then added into wells of a 96-well plate, followed by the addition of PA_20_ to specific wells to a concentration of 1 μg/mL. The bacteria were incubated in 96-well plates at appropriate temperatures with constant shaking. The OD_600_ was determined every 610 seconds for 700 minutes by a microplate reader (Molecular Devices, Spectra Max 384 PLUS).

### PRGP-S pull-down assay

This assay was performed according to the procedure of Yoshida *et al*. [91] with minor modifications. Approximately 1 mg of insoluble dap-type peptidoglycan from *B*. *subtilis* was dispersed in 1 mL of PBS and centrifuged at 12,600 x g for 5 min. This process was repeated three times, and the sedimented peptidoglycan was resuspended in 1 mL of PBS. Ten μL (0.5 mg/mL) of recombinant human PGRP-S was mixed with 100 μg of peptidoglycan suspension or PA_20_, and incubated overnight at 4°C. The mixture was centrifuged at 12,600 x g for 5 min, and the pellets were washed three times with 300 μL PBS and resuspended in 200 μL Laemmli sample buffer (VWR, 89230–104). Both the pellets and the supernatants were analyzed by Western blot using anti-PA_83_ and anti-PGRP-S antibodies.

### PGRP-SA and PRGP-S binding ELISAs

PA_20_, washed dap-type peptidoglycan, or washed lys-type peptidoglycan were diluted to 10 μg/mL in bicarbonate buffer, and 50 μL was added to flat-bottom, high-binding, half-area 96-well plates (Corning, 29442–318) in triplicates and incubated overnight at 4°C. The next day, the wells were rinsed with PBST and PBS, then blocked with 100 μL blocking buffer (5% non-fat milk in PBS (PGRP-SA) or 5% BSA [Roche, 10738328103] in PBS (PGRP-S)) for 2 hours at room temperature. Wells were washed two times with PBST, and two times with PBS. Fifty μL of PRGP-SA with a T7 tag or PGRP-S diluted in PBS to 200 ng/μL was added to each well, and the plates were incubated for 2 hours at room temperature. The wells were washed as previously described, and 50 μL of anti-T7 monoclonal antibody (Sigma) diluted to 6.7 μg/mL in PBST and incubated overnight at 4°C. For the PGRP-S plate, anti-PRGP-S polyclonal antibody diluted to 0.5 μg/mL in 5% BSA added to each well and incubated for 2 hours at room temperature. The wells were washed as above, and horseradish peroxidase-conjugated anti-mouse secondary antibody, diluted in PBST for PGRP-SA and 5% BSA for PGRP-S, was added to each well and incubated for 2 hours at room temperature. Following PBST and PBS washes, 50 μL of *o*-Phenylenediamine dihydrochloride (Sigma, P1526) dissolved in deionized water was added to each well, and the plate was incubated for 30 minutes at room temperature. The absorbance at 450 nm was measured using a microplate reader (Molecular Devices, Spectra Max 384 PLUS).

### Measurement of reactive oxygen species in S2 cells

Reactive oxygen species (ROS) consisting of hydrogen peroxide, peroxynitrite, hydroxyl radicals, nitric oxide, and peroxy radicals, and separately superoxide radicals were measured using ROS-ID Total ROS/Superoxide Detection Kit (Enzo Life Sciences, ENZ-51010). S2 cells (Expression Systems, 94-005F) were collected by centrifugation at 400 x g for 5 minutes and resuspended in ESF 921 medium (Expression Systems, 96-001-01). Cells were seeded at 500,000 cells per well in 50 μL of resuspension medium into clear 96-well plates with black chimneys. Dilutions of PA_20_ were performed in separate 12 channel basins with LB or LB containing *B*. *cereus* overnight culture at 1.6 OD_600_. PA_20_ was tested at 22, 2, 1, 0.5, and 0.25 μg/mL. A condition with no PA_20_ was included with every assay. The oxidative stress detection reagent and the superoxide detection reagent were reconstituted in anhydrous DMF (VWR, 97064–586) to yield 5 mM stock solutions and stored at -20°C. Detection reagents were added at 0.04% of sample preparation. Fifty μL of sample preparation reagent was added to the 96-well containing S2 cells and then incubated at 27°C in the dark for 30 minutes. Fluorescence was measured with bottom reading for two different wavelengths. Total ROS was measured at excitation 488 nm, cutoff 515 nm, and emission 520 nm. Superoxide detection was measured at excitation 550 nm, cutoff 610 nm, and emission 610 nm (Molecular Devices, SpectraMax Gemini XPS/EM Microplate Reader).

### Data analysis

Data analysis was conducted using GraphPad Prism software. All *P*-values reported are products of the respective positive control to a single experimental condition using two statistical analyses: the Log-rank (Mantel-Cox) and the Gehan-Breslow-Wilcoxon tests. An alpha of 0.05 was deemed the threshold for significance. We report *P* values adjusted by the Bonferroni correction. The delay in median survival was reported. Since the chance of dying in a small-time interval was not the same early in the study and late in the study, the values for the 95% CI of the ratio of median survivals were not meaningful and were not reported. Flies that died within the first 24 hours of the assay were censored from statistical analysis and considered to have died due to non-infective causes. Each insect experiment shown is representative of at least three independent experiments.

## Supporting information

S1 Fig The effect of anthrax toxins on the longevity of flies. In the absence of bacterial challenge, oral administration of anthrax toxins does not affect fly survival: flies were fed a 50 mM sucrose solution (WT condition) or a solution containing anthrax toxin components PA_83_, PA_63_, PA_20_, LF and EF, resuspended in 50 mM sucrose.
(TIF)

S2 Fig PA_83_ protects unaged *Drosophila* from *Bacillus cereus*. Male WT flies of various ages were challenged with *B*. *cereus* in the absence or presence of PA_83_. *P* as in Fig 1.
(TIF)

S3 Fig PA_20_ 1β_13_−1α_1_ loop does not alter the growth of *B*. *cereus*. Evaluation of the effects of cortisone and PA_83_ 1β_13_−1α_1_ loop. Agar diffusion susceptibility assay of *B*. *cereus* grown on LB solid medium. Plates were spread with 50 μL of bacterial overnight culture diluted to an OD_600_ of 0.1 and subsequently spotted with 1 μL of the following reagents. The plate was left to incubate overnight at 37 ˚C. Spots below a through e contain a 1 μL spot of: 20 mM cortisone acetate (a), 1 μg/mL of PA_83_ 1β_13_−1α_1_ loop (aa 181–200), (b) 1 μg/mL of PA_20_ 1β_13_−1α_1_ loop (aa 181–192) (c), PBS (d), 10 mM levofloxacin (e).
(TIF)

S4 Fig PA_83_ protects unaged *Drosophila* from *Serratia liquefaciens*. Male WT flies of various ages were challenged with *S*. *liquefaciens* in the absence or presence of PA_83_. *P* as in Fig 1.
(TIF)

S5 Fig The effect of PA_20_ on the sensitivity of fly mutants to *S*. *liquefaciens*. The effect of PA_20_ or PA_83_ on the Toll and Imd pathways mutants. SPE (A), SPZ (B), Imd (C), Dsor1 (with PA_83_) (D), and Dsor1 (with PA_20_) (E) mutant flies were fed a 50 mM sucrose solution in the presence or the absence of *S*. *liquefaciens*, or *S*. *liquefaciens* with 1 μg/mL of PA_20_ or PA_83_. Flies were maintained at 30°C and monitored for death a minimum of twice daily and expressed as percent survival. *P* as in Fig 1.
(TIF)

S6 Fig Determination of minimal immunosuppressive concentration of cortisone acetate. Titration assays revealed that 20 mM cortisone acetate added to the feeding medium was the minimum concentration sufficient to immunosuppress *Drosophila* (Fig 6I), as 10mM did not alter the sensitivity of *Drosophila* to *B*. *cereus*. Wild type flies were fed a 50 mM sucrose solution. Some conditions included *B*. *cereus*, which was resuspended in 50 mM sucrose solution, or a condition containing an additional 10 mM cortisone acetate. Flies were maintained at 30°C and monitored for death a minimum of twice daily and expressed as percent survival.
(TIF)

S7 Fig Determining the effect of PA_20_ on superoxide production in S2 cells. Measuring superoxide radicals in S2 cells in the absence and the presence of PA_20_. Superoxides were measured using ROS-ID Total ROS/Superoxide Detection Kit (Enzo Life Sciences). Superoxides produced by S2 cells were measured in the absence or the presence of *B*. *cereus* and various concentrations of PA_20_ (0.25 to 20 μg/mL).
(TIF)

S8 Fig Determination of the longevity of Dsor1 male and female flies. Dsor1 male and female flies were fed a 50mM sucrose solution (Dsor1 M/F). Flies were maintained at 30°C and monitored for death a minimum of twice daily and expressed as percent survival. Note, no bacterium was included in this experiment.
(TIF)
