# Supplementary figures and images for: Anthrax toxin component, Protective Antigen, protects insects from bacterial infections
Source: PLoS Pathog. 2020 Aug 31;16(8):e1008836. doi: 10.1371/journal.ppat.1008836 (PMC7458312; doi:10.1371/journal.ppat.1008836)

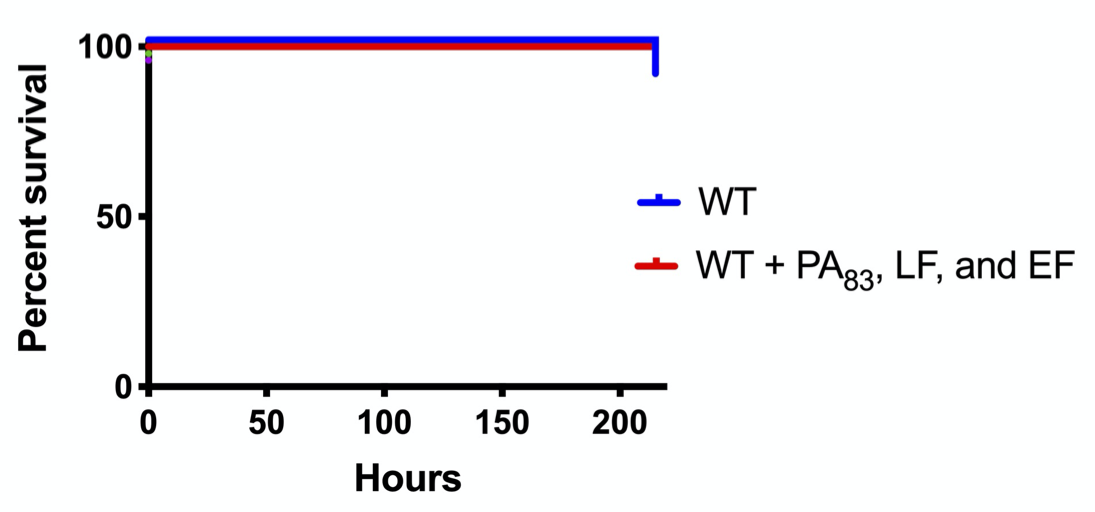

Supplement: S1 Fig — In the absence of bacterial challenge, oral administration of anthrax toxins does not affect fly survival: flies were fed a 50 mM sucrose solution (WT condition) or a solution containing anthrax toxin components PA83, PA63, PA20, LF and EF, resuspended in 50 mM sucrose. (TIF) [file ppat.1008836.s001.tif]

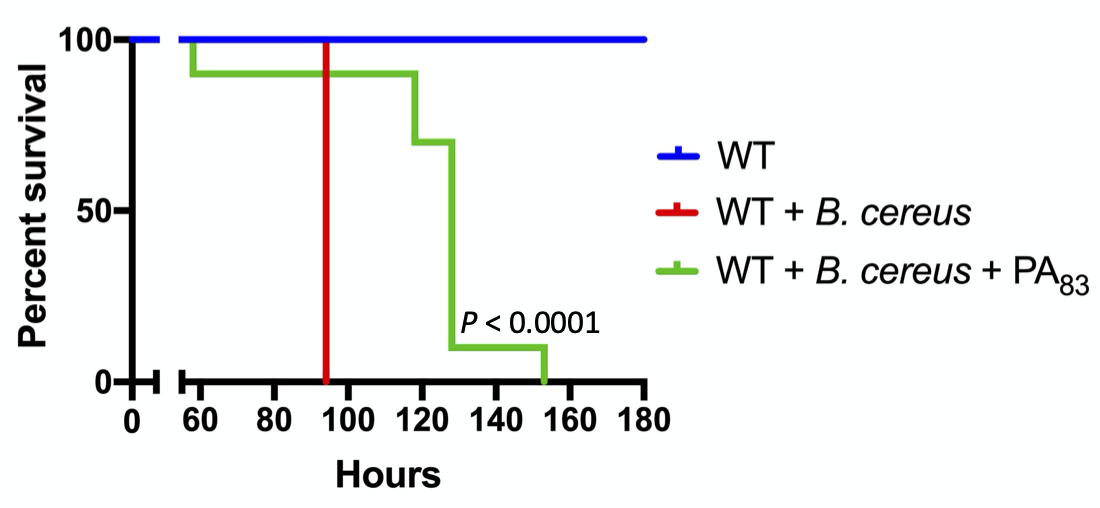

Supplement: S2 Fig — Male WT flies of various ages were challenged with B. cereus in the absence or presence of PA83. P as in Fig 1. (TIF) [file ppat.1008836.s002.tif]

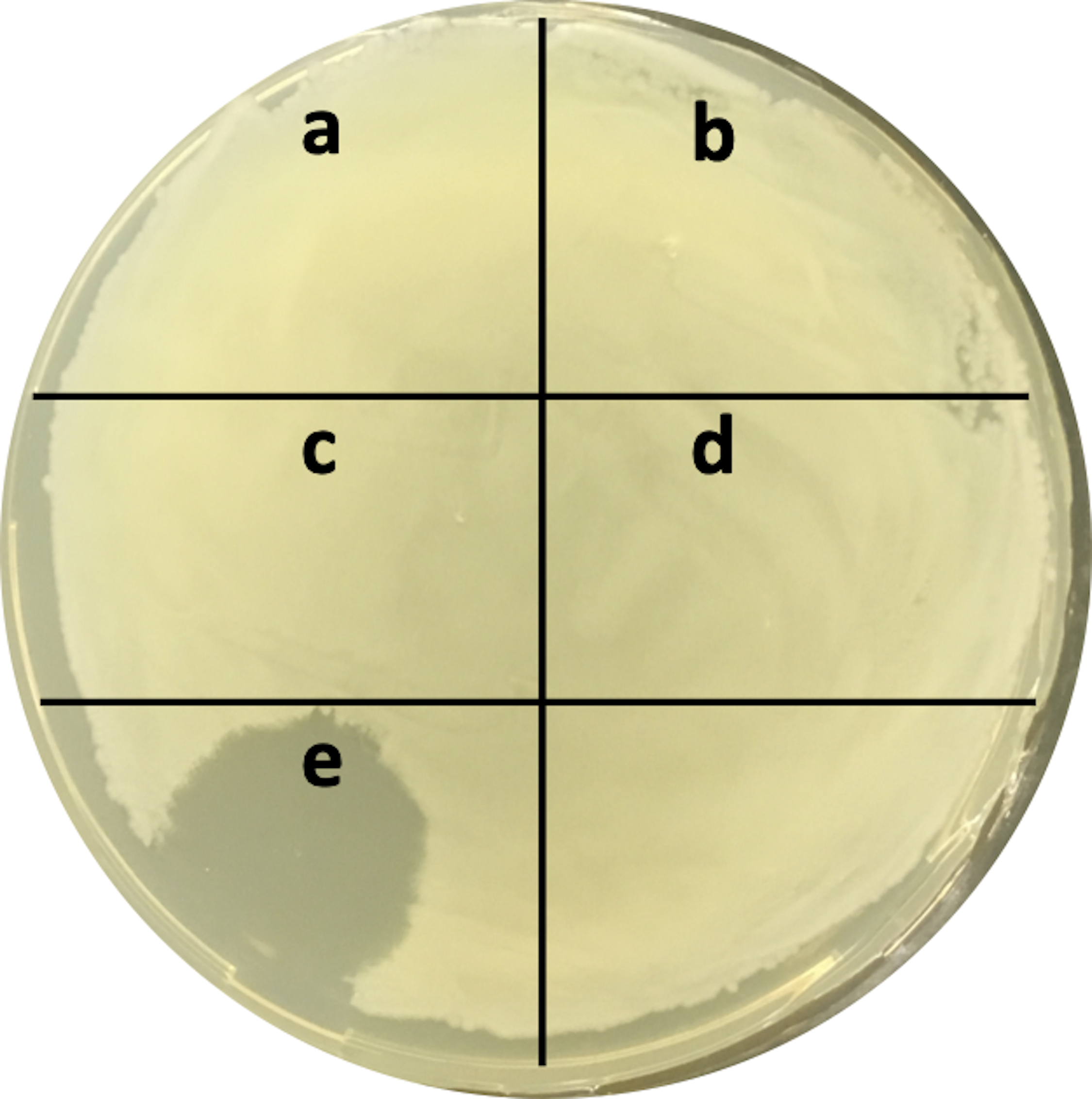

Supplement: S3 Fig — Evaluation of the effects of cortisone and PA83 1β13−1α1 loop. Agar diffusion susceptibility assay of B. cereus grown on LB solid medium. Plates were spread with 50 μL of bacterial overnight culture diluted to an OD600 of 0.1 and subsequently spotted with 1 μL of the following reagents. The plate was left to incubate overnight at 37 ˚C. Spots below a through e contain a 1 μL spot of: 20 mM cortisone acetate (a), 1 μg/mL of PA83 1β13−1α1 loop (aa 181–200), (b) 1 μg/mL of PA20 1β13−1α1 loop (aa 181–192) (c), PBS (d), 10 mM levofloxacin (e). (TIF) [file ppat.1008836.s003.tif]

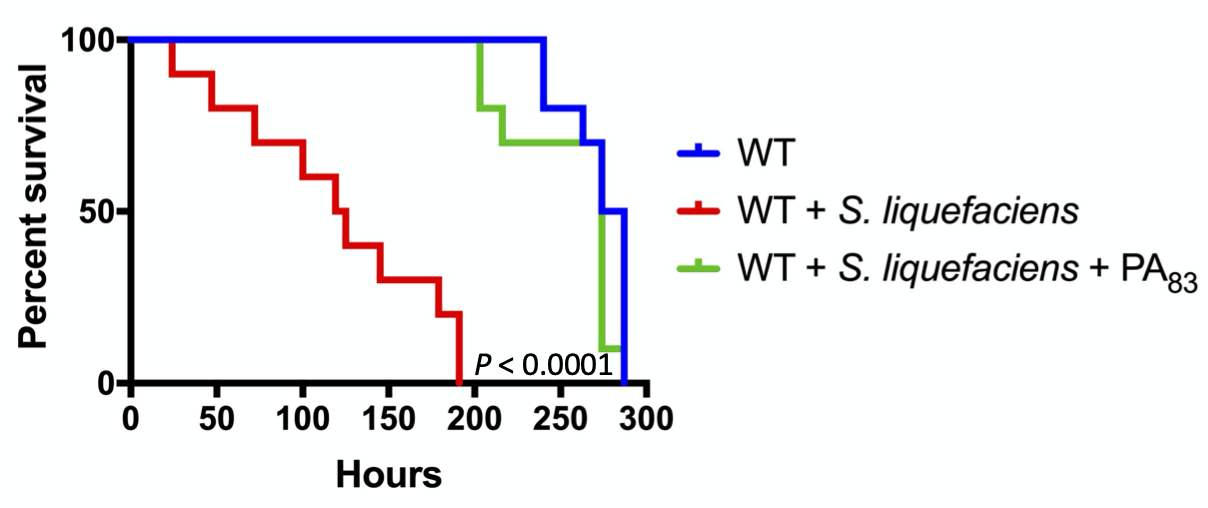

Supplement: S4 Fig — Male WT flies of various ages were challenged with S. liquefaciens in the absence or presence of PA83. P as in Fig 1. (TIF) [file ppat.1008836.s004.tif]

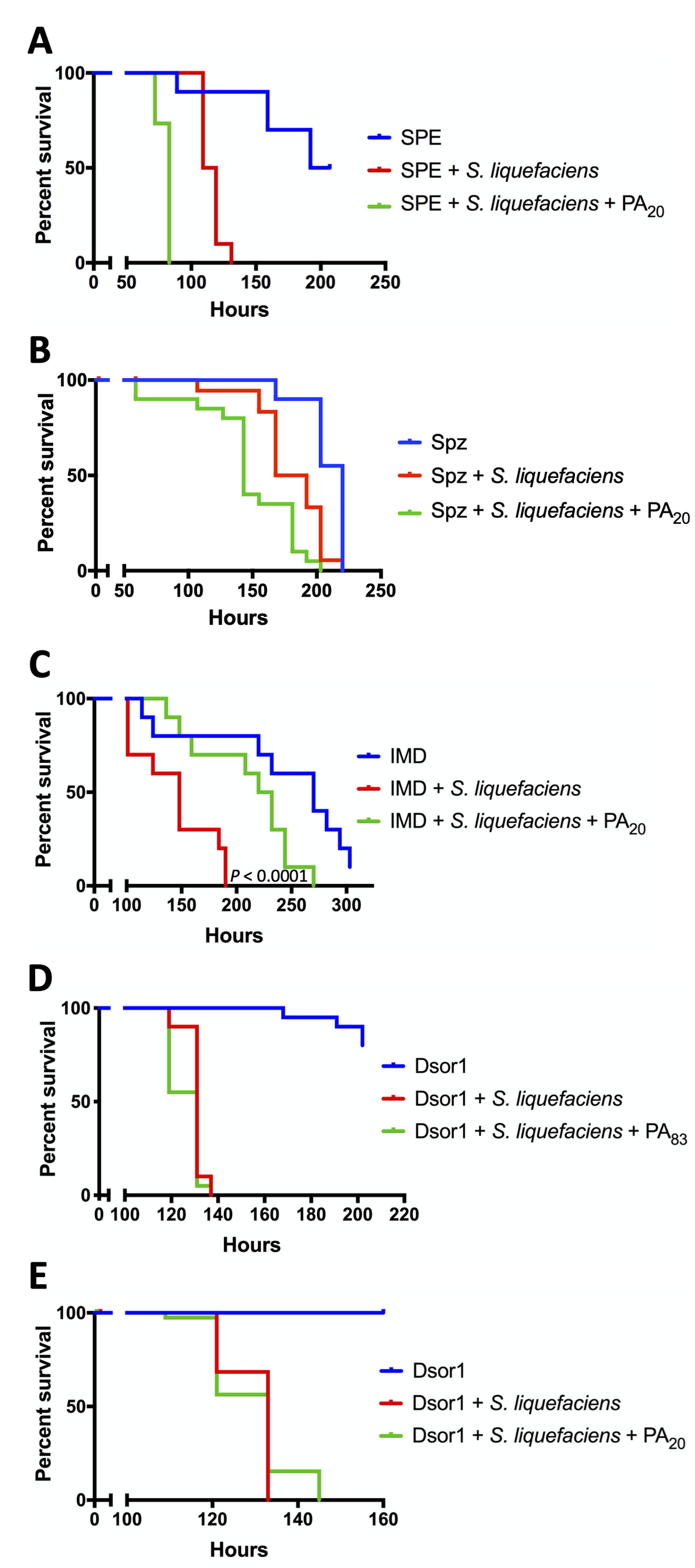

Supplement: S5 Fig — The effect of PA20 or PA83 on the Toll and Imd pathways mutants. SPE (A), SPZ (B), Imd (C), Dsor1 (with PA83) (D), and Dsor1 (with PA20) (E) mutant flies were fed a 50 mM sucrose solution in the presence or the absence of S. liquefaciens, or S. liquefaciens with 1 μg/mL of PA20 or PA83. Flies were maintained at 30°C and monitored for death a minimum of twice daily and expressed as percent survival. P as in Fig 1. (TIF) [file ppat.1008836.s005.tif]

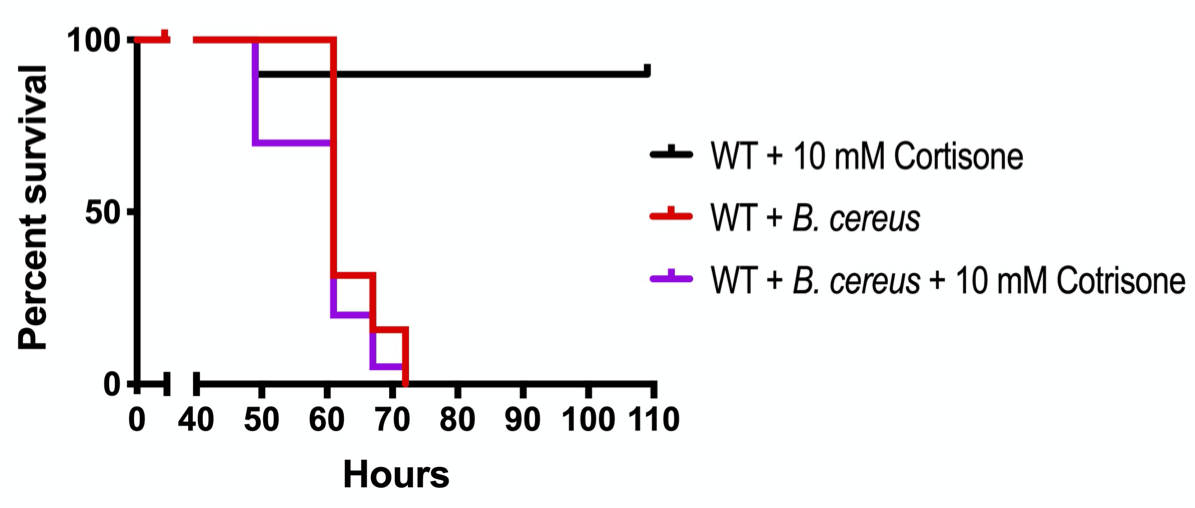

Supplement: S6 Fig — Titration assays revealed that 20 mM cortisone acetate added to the feeding medium was the minimum concentration sufficient to immunosuppress Drosophila (Fig 6I), as 10mM did not alter the sensitivity of Drosophila to B. cereus. Wild type flies were fed a 50 mM sucrose solution. Some conditions included B. cereus, which was resuspended in 50 mM sucrose solution, or a condition containing an additional 10 mM cortisone acetate. Flies were maintained at 30°C and monitored for death a minimum of twice daily and expressed as percent survival. (TIF) [file ppat.1008836.s006.tif]

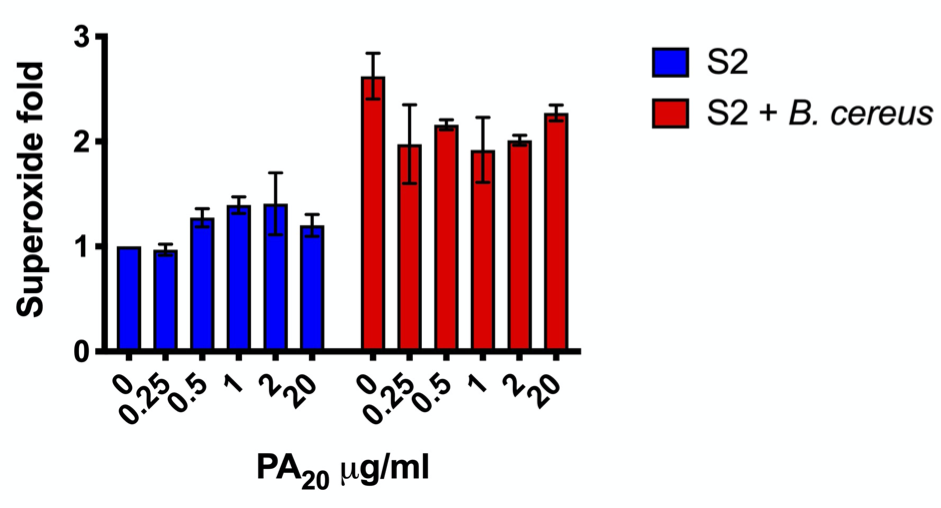

Supplement: S7 Fig — Measuring superoxide radicals in S2 cells in the absence and the presence of PA20. Superoxides were measured using ROS-ID Total ROS/Superoxide Detection Kit (Enzo Life Sciences). Superoxides produced by S2 cells were measured in the absence or the presence of B. cereus and various concentrations of PA20 (0.25 to 20 μg/mL). (TIF) [file ppat.1008836.s007.tif]

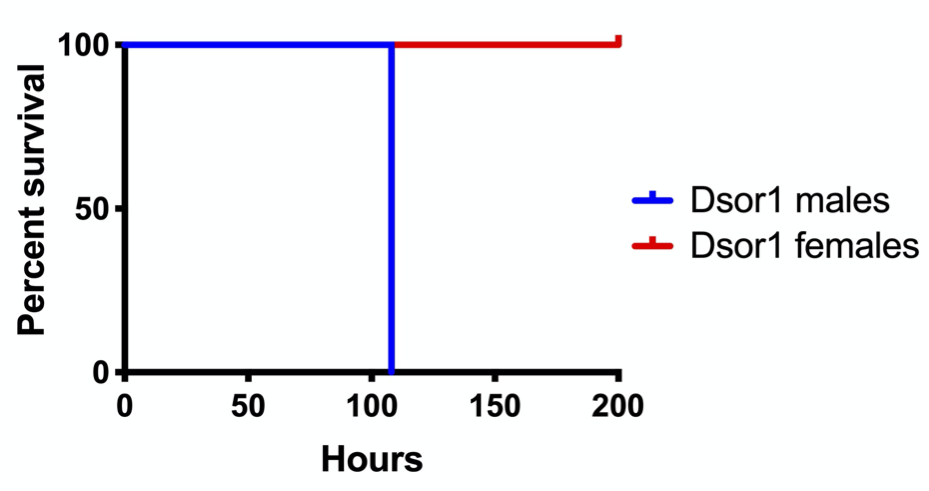

Supplement: S8 Fig — Dsor1 male and female flies were fed a 50mM sucrose solution (Dsor1 M/F). Flies were maintained at 30°C and monitored for death a minimum of twice daily and expressed as percent survival. Note, no bacterium was included in this experiment. (TIF) [file ppat.1008836.s008.tif]
